# Supplementary material for: Continuity Scaling: A Rigorous Framework for Detecting and Quantifying Causality Accurately
Source: Research (Wash D C). 2022 May 4;2022:9870149. doi: 10.34133/2022/9870149 (PMC9101326; doi:10.34133/2022/9870149)
Supplement: Supplementary Materials — SI.pdf (where we include analytic and computational details of the results in the main text. This SI is helpful but not essential for understanding the main results of the paper.) [file 9870149.f1.zip › SI.pdf]

Supplementary Information for "Continuity scaling: A rigorous framework for detecting and quantifying causality accurately"

Xiong Ying,[1,2,3] Si-Yang Leng,[2,4] Huan-Fei Ma,[5]
Qing Nie,[6] Ying-Cheng Lai,[7] and Wei Lin[1,2,3,8,*]

[1]*School of Mathematical Sciences, SCMS, and SCAM, Fudan University, Shanghai 200433, China*
[2]*Research Institute for Intelligent Complex Systems, CCSB, and LCNBI, Fudan University, Shanghai 200433, China*
[3]*State Key Laboratory of Medical Neurobiology, and MOE Frontiers Center for Brain Science, Institutes of Brain Science, Fudan University, Shanghai 200032, China*
[4]*Institute of AI and Robotics, Academy for Engineering and Technology, Fudan University, Shanghai 200433, China*
[5]*School of Mathematical Sciences, Soochow University, Suzhou 215006, China*
[6]*Department of Mathematics, Department of Developmental and Cell Biology, and NSF-Simons Center for Multiscale Cell Fate Research, University of California, Irvine, CA 92697-3875, USA*
[7]*School of Electrical, Computer, and Energy Engineering, Arizona State University, Tempe, Arizona 85287-5706, USA*
[8]*Shanghai Artificial Intelligence Laboratory, Shanghai 200232, China*

(Dated: March 23, 2022)

Abstract

The Supplementary Information provides analytic and computational details of the results in the main text. It is helpful but not essential for understanding the main results of the paper. The materials are organized as

CONTENTS

I. Mathematical notations 3

II. Estimating radius of neighborhood and scaling law 3

III. Additional examples demonstrating the power of continuity scaling framework in ascertaining and quantifying causation 8
- A. Unidirectionally and bidirectionally coupled systems 8
  - 1. Coupled ecological models 8
  - 2. Coupled Lorenz systems 9
  - 3. Coupled Rössler-Lorenz systems 15
- B. Effects of varying sampling duration 15
- C. Additional examples with complex nonlinear coupling schemes 16
- D. Inferring networks of causal interactions 20
- E. Test of real-world datasets 21
  - 1. Synthetic gene regulatory networks 21
  - 2. Fishery landings and sea surface temperature data 22
  - 3. World COVID-19 pandemic daily cases 23
  - 4. Air pollutants and hospital admission records from Hong Kong 26

IV. Comparisons with typical cross-map-based methods 27
- A. Comparison with topological causality 27
- B. Comparison with convergent cross mapping 29

References 32

* wlin@fudan.edu.cn

I. MATHEMATICAL NOTATIONS

Listed in Tab. S1 are mathematical notations used in this work.

TABLE S1. Illustrations on notations.

Notations	Illustrations
$\boldsymbol{x}_t,\ \boldsymbol{y}_t$	Internal states of the original dynamical system at time t evolving on some compact manifold.
$u(\cdot),\ v(\cdot)$	Observational functions, either depending on only partial Cartesian coordinates of the state variables $\boldsymbol{x}$ and $\boldsymbol{y}$, respectively, or containing mixed information from the state variables.
$u_t,\ v_t$	Values of the observational functions from the internal states of the original dynamical system at time t.
$\boldsymbol{u}_t,\ \boldsymbol{v}_t$	Reconstructed state vectors at time t consisting of the delayed coordinates based on the observational time series $\{u_t\}_{t\in\mathbb{N}}$ and $\{v_t\}_{t\in\mathbb{N}}$, respectively.
$\varepsilon_{\boldsymbol{u}}$	Radius of the neighborhood on the manifold where the variable $\boldsymbol{u}$ is assumed to represent an effect.
$\delta^t_{\boldsymbol{v}}(\varepsilon_{\boldsymbol{u}})$	Radius of the pull-back neighborhood on the manifold where $\boldsymbol{v}$ is supposed to be a causal variable, which corresponds to the neighborhood of radius $\varepsilon_{\boldsymbol{u}}$. Here, time t indicates the point at which the estimation of the radius is applicable.
$\langle\delta^t_{\boldsymbol{v}}(\varepsilon_{\boldsymbol{u}})\rangle_{t\in\mathbb{N}}$	The average of $\delta^t_{\boldsymbol{v}}(\varepsilon_{\boldsymbol{u}})$ over all possible time t for a given number $\varepsilon_{\boldsymbol{u}}$.
$s_{\boldsymbol{v}\hookrightarrow\boldsymbol{u}}$	Estimated slope of the regression line obtained from the scaling relation between $\langle\delta^t_{\boldsymbol{v}}(\varepsilon_{\boldsymbol{u}})\rangle_t$ and $\ln\varepsilon_{\boldsymbol{u}}$.

II. ESTIMATING RADIUS OF NEIGHBORHOOD AND SCALING LAW

We rewrite the considered original dynamical system here as

$$\boldsymbol{x}_{t+1} = \boldsymbol{f}(\boldsymbol{x}_t, \boldsymbol{y}_t), \quad \boldsymbol{y}_{t+1} = \boldsymbol{g}(\boldsymbol{x}_t, \boldsymbol{y}_t), \quad t \in \mathbb{N}, \tag{S2.1}$$

where $\{\boldsymbol{x}_t\}_{t\in\mathbb{N}}$ and $\{\boldsymbol{y}_t\}_{t\in\mathbb{N}}$ evolve on compact manifolds $\mathcal{M}$, $\mathcal{N}$ of dimension $D_{\mathcal{M}}$, $D_{\mathcal{N}}$ respectively and $[\boldsymbol{f}, \boldsymbol{g}]$ are sufficiently smooth map functions. The internal states $\{\boldsymbol{x}_t, \boldsymbol{y}_t\}_{t\in\mathbb{N}}$ of system (S2.1) are either completely or partially accessible and $\{u_t, v_t\}_{t\in\mathbb{N}}$ are the two observational time series. Let $u_t = u(\boldsymbol{x}_t)$ and $v_t = v(\boldsymbol{y}_t)$. With the embedding dimensions $d_{u,v}$ and delay time $\tau_{u,v}$ properly chosen according to some empirical criteria (See Methods in the main text), we reconstruct the state vectors as $\boldsymbol{z}_t = (z_t, z_{t+\tau_z}, \dots, z_{t+(d_z-1)\tau_z})$, where $\boldsymbol{z} = \boldsymbol{u}, \boldsymbol{v}$ and $z = u, v$. The dynamical evolution of the reconstructed state vectors is governed by

$$\boldsymbol{u}_{t+1} = \tilde{\boldsymbol{f}}(\boldsymbol{u}_t, \boldsymbol{v}_t), \quad \boldsymbol{v}_{t+1} = \tilde{\boldsymbol{g}}(\boldsymbol{u}_t, \boldsymbol{v}_t), \quad t \in \mathbb{N}, \tag{S2.2}$$

where $\boldsymbol{u}_t \in \mathcal{L}_u$ and $\boldsymbol{v}_t \in \mathcal{L}_v$. When the observational functions so defined are identity functions, the explicit intrinsic states of the original system are recovered: $\boldsymbol{u} = \boldsymbol{x}$ and

$\boldsymbol{v} = \boldsymbol{y}$. The mathematical reasoning below is thus not only suitable for the more general Case II defined in the main text, but also for Case I through a direct substitution of $\boldsymbol{u}$ and $\boldsymbol{v}$ by $\boldsymbol{x}$ and $\boldsymbol{y}$, respectively.

For a given $\boldsymbol{u}_g \in \mathcal{L}_u$, we set $\tilde{\boldsymbol{f}}_{\boldsymbol{u}_g}(\cdot) \triangleq \tilde{\boldsymbol{f}}(\boldsymbol{u}_g, \cdot)$. For any given $\varepsilon_{\boldsymbol{u}} > 0$, we examine the preimage of the neighborhood $\mathcal{O}(\boldsymbol{u}_{t+1}, \varepsilon_{\boldsymbol{u}})$. Denoting this pull-back neighborhood as $\tilde{\boldsymbol{f}}_{\boldsymbol{u}_t}^{-1}(\mathcal{O}(\boldsymbol{u}_{t+1}, \varepsilon_{\boldsymbol{u}}))$, we have that $\tilde{\boldsymbol{f}}_{\boldsymbol{u}_t}^{-1}(\mathcal{O}(\boldsymbol{u}_{t+1}, \varepsilon_{\boldsymbol{u}}))$ is a neighborhood of $\boldsymbol{v}_t$ because of the relation $\tilde{\boldsymbol{f}}_{\boldsymbol{u}_t}(\boldsymbol{v}_t) = \boldsymbol{u}_{t+1}$ from system (S2.2). Of particular interest is *whether and how* $\tilde{\boldsymbol{f}}_{\boldsymbol{u}_t}^{-1}(\mathcal{O}(\boldsymbol{u}_{t+1}, \varepsilon_{\boldsymbol{u}}))$ contracts to the point $\boldsymbol{v}_t$ as $\varepsilon_{\boldsymbol{u}}$ tends to zero, as the existence of some scaling of this correspondent continuity is not only indicative but also a quantitative characterization of causation from $\boldsymbol{v}_t$ to $\boldsymbol{u}_t$ at time t.

To obtain the continuity scaling, for given $\varepsilon_{\boldsymbol{u}}$, we analytically define the "radius" of the neighborhood $\tilde{\boldsymbol{f}}_{\boldsymbol{u}_t}^{-1}(\mathcal{O}(\boldsymbol{u}_{t+1}, \varepsilon_{\boldsymbol{u}}))$ as

$$\delta_{\boldsymbol{v}}^t(\varepsilon_{\boldsymbol{u}}) \triangleq \left\{\mathrm{Vol}\left[\tilde{\boldsymbol{f}}_{\boldsymbol{u}_t}^{-1}(\mathcal{O}(\boldsymbol{u}_{t+1}, \varepsilon_{\boldsymbol{u}}))\right]\right\}^{-1} \int_{\tilde{\boldsymbol{f}}_{\boldsymbol{u}_t}^{-1}(\mathcal{O}(\boldsymbol{u}_{t+1}, \varepsilon_{\boldsymbol{u}}))} \mathrm{dist}_{\mathcal{L}_v}(\boldsymbol{v}, \boldsymbol{v}_t)\, \mathrm{d}\boldsymbol{v}, \tag{S2.3}$$

where $\mathrm{Vol}[\cdot]$ represents the volume of a given set. In applications, one relies on the reconstructed measured time series $\{\boldsymbol{u}_t, \boldsymbol{v}_t\}_{t\in\mathbb{N}}$ to estimate the quantity $\delta_{\boldsymbol{v}}^t(\varepsilon_{\boldsymbol{u}})$. The most accurate estimation obtained in an ideal situation is

$$\delta_{\boldsymbol{v}}^t(\varepsilon_{\boldsymbol{u}}) \triangleq \left\{\#\hat{I}(\varepsilon_{\boldsymbol{u}})\right\}^{-1} \sum_{\iota\in\hat{I}(\varepsilon_{\boldsymbol{u}})} \mathrm{dist}_{\mathcal{L}_v}(\boldsymbol{v}_t, \boldsymbol{v}_\iota), \tag{S2.4}$$

where

$$\hat{I}(\varepsilon_{\boldsymbol{u}}) \triangleq \left\{\iota \in \mathbb{N} \,\middle|\, \boldsymbol{v}_\iota \in \tilde{\boldsymbol{f}}_{\boldsymbol{u}_t}^{-1}(\mathcal{O}(\boldsymbol{u}_{t+1}, \varepsilon_{\boldsymbol{u}}))\right\} \tag{S2.5}$$

and $\#[\cdot]$ is the cardinality of a given set. However, because of lack of sufficient information about $\tilde{\boldsymbol{f}}$, in general such an estimation cannot be obtained directly. The ideal index set $\hat{I}(\varepsilon_{\boldsymbol{u}})$ defined in Eq. (S2.5) thus is usually not available.

To overcome this difficulty, we make use of the following

$$\sup_{\boldsymbol{v}\in\mathcal{L}_v} \mathrm{dist}_{\mathcal{L}_u}(\tilde{\boldsymbol{f}}_{\boldsymbol{u}'}(\boldsymbol{v}), \tilde{\boldsymbol{f}}_{\boldsymbol{u}''}(\boldsymbol{v})) \to 0, \text{ as } \mathrm{dist}_{\mathcal{L}_u}(\boldsymbol{u}', \boldsymbol{u}'') \to 0, \tag{S2.6}$$

which is the result of uniform continuity of $\tilde{\boldsymbol{f}}$ on the compact manifold $\mathcal{L}_u \times \mathcal{L}_v$. Another fact is

$$\mathrm{dist}_{\mathcal{L}_u}(\boldsymbol{u}_{t+1}, \tilde{\boldsymbol{f}}_{\boldsymbol{u}_t}(\boldsymbol{v}_\iota)) = \mathrm{dist}_{\mathcal{L}_u}(\tilde{\boldsymbol{f}}_{\boldsymbol{u}_t}(\boldsymbol{v}_t), \tilde{\boldsymbol{f}}_{\boldsymbol{u}_t}(\boldsymbol{v}_\iota)). \tag{S2.7}$$

The triangle inequality gives

$$\left|\mathrm{dist}_{\mathcal{L}_u}(\tilde{\boldsymbol{f}}_{\boldsymbol{u}_t}(\boldsymbol{v}_\iota), \tilde{\boldsymbol{f}}_{\boldsymbol{u}_t}(\boldsymbol{v}_t)) - \mathrm{dist}_{\mathcal{L}_u}(\tilde{\boldsymbol{f}}_{\boldsymbol{u}_t}(\boldsymbol{v}_t), \tilde{\boldsymbol{f}}_{\boldsymbol{u}_\iota}(\boldsymbol{v}_\iota))\right| \leqslant \mathrm{dist}_{\mathcal{L}_u}(\tilde{\boldsymbol{f}}_{\boldsymbol{u}_t}(\boldsymbol{v}_\iota), \tilde{\boldsymbol{f}}_{\boldsymbol{u}_\iota}(\boldsymbol{v}_\iota)) \tag{S2.8}$$

which, from Eq. (S2.6), approaches zero as $\mathrm{dist}_{\mathcal{L}_u}(\boldsymbol{u}_t, \boldsymbol{u}_\iota)$ tends to zero. Applying Eqs. (S2.6) and (S2.7) as well as the inequality (S2.8), we get

$$\begin{aligned} &\left\{\boldsymbol{v}_\iota \in \mathcal{L}_v \,\middle|\, \boldsymbol{v}_\iota \in \tilde{\boldsymbol{f}}_{\boldsymbol{u}_t}^{-1}(\mathcal{O}(\boldsymbol{u}_{t+1}, \varepsilon_{\boldsymbol{u}}))\right\} \\ &\qquad = \left\{\boldsymbol{v}_\iota \in \mathcal{L}_v \,\middle|\, \mathrm{dist}_{\mathcal{L}_u}(\boldsymbol{u}_{t+1}, \tilde{\boldsymbol{f}}_{\boldsymbol{u}_t}(\boldsymbol{v}_\iota)) < \varepsilon_{\boldsymbol{u}}\right\} \\ &\qquad = \left\{\boldsymbol{v}_\iota \in \mathcal{L}_v \,\middle|\, \mathrm{dist}_{\mathcal{L}_u}(\tilde{\boldsymbol{f}}_{\boldsymbol{u}_t}(\boldsymbol{v}_t), \tilde{\boldsymbol{f}}_{\boldsymbol{u}_t}(\boldsymbol{v}_\iota)) < \varepsilon_{\boldsymbol{u}}\right\} \\ &\qquad \approx \left\{\boldsymbol{v}_\iota \in \mathcal{L}_v \,\middle|\, \mathrm{dist}_{\mathcal{L}_u}(\tilde{\boldsymbol{f}}_{\boldsymbol{u}_t}(\boldsymbol{v}_t), \tilde{\boldsymbol{f}}_{\boldsymbol{u}_\iota}(\boldsymbol{v}_\iota)) < \varepsilon_{\boldsymbol{u}}\right\} \\ &\qquad = \left\{\boldsymbol{v}_\iota \in \mathcal{L}_v \,\middle|\, \mathrm{dist}_{\mathcal{L}_u}(\boldsymbol{u}_{t+1}, \boldsymbol{u}_{\iota+1}) < \varepsilon_{\boldsymbol{u}}\right\}, \end{aligned} \tag{S2.9}$$

provided that $\mathrm{dist}_{\mathcal{L}_u}(\boldsymbol{u}_t, \boldsymbol{u}_\iota)$ is sufficiently small. Letting $\iota = \tau - 1$ yields a new setting for the radius estimation as

$$\delta_{\boldsymbol{v}}^t(\varepsilon_{\boldsymbol{u}}) \triangleq \left\{\#[\bar{I}_{\boldsymbol{u}}^t(\varepsilon_{\boldsymbol{u}})]\right\}^{-1} \sum_{\tau \in \bar{I}_{\boldsymbol{u}}^t(\varepsilon_{\boldsymbol{u}})} \mathrm{dist}_{\mathcal{L}_v}(\boldsymbol{v}_t, \boldsymbol{v}_{\tau-1}), \tag{S2.10}$$

where

$$\bar{I}_{\boldsymbol{u}}^t(\varepsilon_{\boldsymbol{u}}) \triangleq \left\{\tau \in \mathbb{N} \;\middle|\; \mathrm{dist}_{\mathcal{L}_u}(\boldsymbol{u}_t, \boldsymbol{u}_{\tau-1}) < \varepsilon_{\boldsymbol{u}},\ \mathrm{dist}_{\mathcal{L}_u}(\boldsymbol{u}_{t+1}, \boldsymbol{u}_\tau) < \varepsilon_{\boldsymbol{u}}\right\} \tag{S2.11}$$

has been used to approximate the set $\hat{I}_{\boldsymbol{u}}^t(\varepsilon_{\boldsymbol{u}})$ specified in Eq. (S2.5). This indicates that the scaling relation at time t between $\delta_{\boldsymbol{v}}^t(\varepsilon_{\boldsymbol{u}})$ and $\ln \varepsilon_{\boldsymbol{u}}$, which describes the changes of the set $\tilde{\boldsymbol{f}}_{\boldsymbol{u}_t}^{-1}\big(\mathcal{O}(\boldsymbol{u}_{t+1}, \varepsilon_{\boldsymbol{u}}) \cap \{\boldsymbol{u}_\tau; \mathrm{dist}_{\mathcal{L}_u}(\boldsymbol{u}_t, \boldsymbol{u}_{\tau-1}) < \varepsilon_{\boldsymbol{u}}\}\big)$ with the contraction of the set $\mathcal{O}(\boldsymbol{u}_{t+1}, \varepsilon_{\boldsymbol{u}}) \cap \{\boldsymbol{u}_\tau; \mathrm{dist}_{\mathcal{L}_u}(\boldsymbol{u}_t, \boldsymbol{u}_{\tau-1}) < \varepsilon_{\boldsymbol{u}}\}$, measures the instantaneous causation from $\boldsymbol{v}$ to $\boldsymbol{u}$ at time t. When $\boldsymbol{u}$ and $\boldsymbol{v}$ are substituted, respectively, by $\boldsymbol{x}$ and $\boldsymbol{y}$, the quantity set in (S2.10) reduces to the quantity in Eq. (2) in the main text.

If the following condition is assumed,

$$\mathrm{dist}_{\mathcal{L}_u}(\boldsymbol{u}_{t+1}, \boldsymbol{u}_\tau) \to 0 \text{ implies } \mathrm{dist}_{\mathcal{L}_u}(\boldsymbol{u}_t, \boldsymbol{u}_{\tau-1}) \to 0, \quad \text{[DD]}$$

the index set $\bar{I}_{\boldsymbol{u}}^t(\varepsilon_{\boldsymbol{u}})$ as defined in (S2.11) can be further simplified as:

$$I_{\boldsymbol{u}}^t(\varepsilon_{\boldsymbol{u}}) \triangleq \left\{\tau \in \mathbb{N} \;\middle|\; \mathrm{dist}_{\mathcal{L}_u}(\boldsymbol{u}_{t+1}, \boldsymbol{u}_\tau) < \varepsilon_{\boldsymbol{u}}\right\}. \tag{S2.12}$$

Consequently, the estimation of the radius of the neighborhood becomes

$$\delta_{\boldsymbol{v}}^t(\varepsilon_{\boldsymbol{u}}) \triangleq \left\{\#[I_{\boldsymbol{u}}^t(\varepsilon_{\boldsymbol{u}})]\right\}^{-1} \sum_{\tau \in I_{\boldsymbol{u}}^t(\varepsilon_{\boldsymbol{u}})} \mathrm{dist}_{\mathcal{L}_v}(\boldsymbol{v}_t, \boldsymbol{v}_{\tau-1}). \tag{S2.13}$$

Since the scaling relation at time t between $\delta_{\boldsymbol{v}}^t(\varepsilon_{\boldsymbol{u}})$ and $\ln \varepsilon_{\boldsymbol{u}}$ is calculated for the situation where $\varepsilon_{\boldsymbol{u}}$ is close to 0, estimation (S2.13) with the simplified index set (S2.12) is applicable to the case where Condition [DD] is fulfilled. In applications, Condition [DD] is often warranted when delayed coordinates are used to reconstruct the states from the observational time series. The examples and simulations shown in Fig. S1 demonstrate the universality of Condition [DD].

The scaling relation, computed by either (S2.10) or (S2.13), represents a characterization at one time instant. To quantify the causation from $\boldsymbol{v}$ to $\boldsymbol{u}$ on the whole manifolds, we use the slope of the regression line obtained from the scaling relation between the averaged $\langle \delta_{\boldsymbol{v}}^t(\varepsilon_{\boldsymbol{u}}) \rangle_{t \in \mathbb{N}}$ and $\ln \varepsilon_{\boldsymbol{u}}$.

The scaling relation between $\langle \delta_{\boldsymbol{v}}^t(\varepsilon_{\boldsymbol{u}}) \rangle_{t \in \mathbb{N}}$ and $\ln \varepsilon_{\boldsymbol{u}}$ can also be analytically demonstrated as the following reasonings, which represents consistency with the numerical simulations. Here we consider the reconstructed system (S2.2). For any given $\varepsilon_{\boldsymbol{u}} > 0$, any $\boldsymbol{u}^* \in \mathcal{O}(\boldsymbol{u}_{t+1}, \varepsilon_{\boldsymbol{u}})$, and $\boldsymbol{v}^*$ close to $\boldsymbol{v}_t$, the Taylor expansion of $\tilde{\boldsymbol{f}}$ to the first order gives

$$\begin{aligned} \boldsymbol{u}_{t+1} - \boldsymbol{u}^* &= \tilde{\boldsymbol{f}}(\boldsymbol{u}_t, \boldsymbol{v}_t) - \boldsymbol{u}^* \\ &\approx \tilde{\boldsymbol{f}}(\boldsymbol{u}_t, \boldsymbol{v}^*) - \boldsymbol{u}^* + \frac{\partial \tilde{\boldsymbol{f}}}{\partial \boldsymbol{v}}(\boldsymbol{u}_t, \boldsymbol{v}^*)(\boldsymbol{v}_t - \boldsymbol{v}^*). \end{aligned} \tag{S2.14}$$

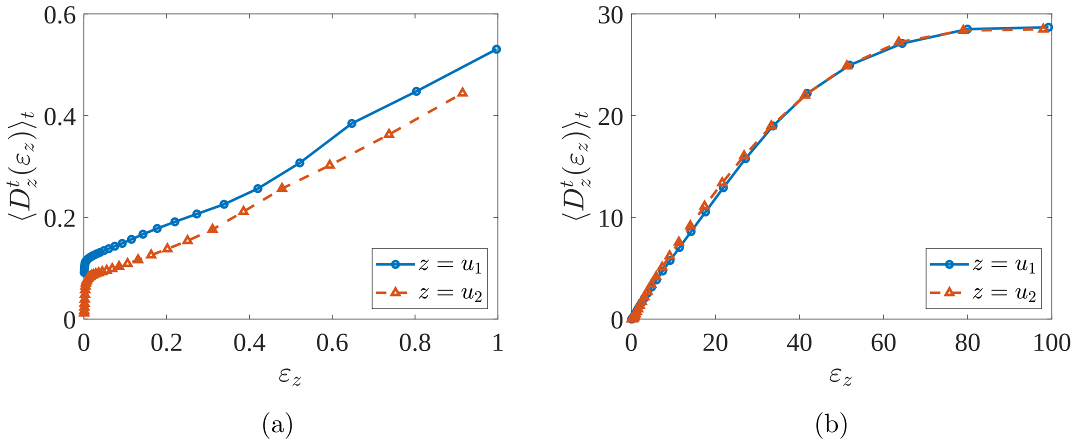

FIG. S1. Two examples demonstrating the universality of Condition [DD]: $\mathrm{dist}(\boldsymbol{z}_{t+1}, \boldsymbol{z}_\tau)\to 0$ implies $\mathrm{dist}(\boldsymbol{z}_t, \boldsymbol{z}_{\tau-1})\to 0$. The notation $\mathrm{dist}(\cdot,\cdot)$ stands for the Euclidean distance in the underlying space and $\boldsymbol{z}$ represents the relevant variable. For a given $\varepsilon_{\boldsymbol{z}} > 0$, Condition [DD] is verified through estimating the average value defined by $D^t_{\boldsymbol{z}}(\varepsilon_{\boldsymbol{z}}) \triangleq (\#[J^t_{\boldsymbol{z}}(\varepsilon_{\boldsymbol{z}})])^{-1}\sum_{\tau\in J^t_{\boldsymbol{z}}(\varepsilon_{\boldsymbol{z}})} \mathrm{dist}(\boldsymbol{z}_t, \boldsymbol{z}_{\tau-1})$, where the index set satisfies $J^t_{\boldsymbol{z}}(\varepsilon_{\boldsymbol{z}}) \triangleq \{\tau \in \mathbb{N} \mid \mathrm{dist}(\boldsymbol{z}_{t+1},\ \boldsymbol{z}_\tau) < \varepsilon_{\boldsymbol{z}},\ |t{+}1{-}\tau| > E\}$, and E is a positive threshold to avoid the situation where the nearest neighboring points are induced only by the consecutive time order. In numerical simulations, $\varepsilon_{\boldsymbol{z}}$ alters its value successively from the set $\{\varepsilon_{\boldsymbol{z},j}\}_{j=1,\cdots,N_\varepsilon}$, where $\varepsilon_{\boldsymbol{z},1} = eD_z$, $\varepsilon_{\boldsymbol{z},N_\epsilon} = D_z$ and $(\ln\varepsilon_{\boldsymbol{z},j} - \ln\varepsilon_{\boldsymbol{z},1})/(j-1) = (\ln\varepsilon_{\boldsymbol{z},N_\varepsilon} - \ln\varepsilon_{\boldsymbol{z},1})/(N_\varepsilon - 1)$, $j = 2, \ldots, N_\varepsilon - 1$. Parameter values are $N_\varepsilon = 33$, $e = 0.001$, and D_z, analogous to Eq. (2) in the main text, is the largest diameter estimated for the underlying manifold. **(a)** The two-species, unidirectionally coupled ecological system (S3.1) with $(r_1, r_2) = (3.8, 3.7)$ and the coupling coefficients $\mu_{21} = 0.05$ and $\mu_{12} = 0$. The system generates time series of 5400 points, where the first 400 points are abandoned to get rid of the transient behavior. The observational functions are $u_i(x_i) = x_i$ and the embedding parameters are $d_i = 3$ and $\tau_i = 1$ so that $\boldsymbol{u}_{i,t} = (u_{i,t}, u_{i,t+1}, u_{i,t+2})$ for $i = 1, 2$, $\boldsymbol{z} = \boldsymbol{u}_{1,2}$, and $E = 0$. **(b)** Unidirectionally coupled Lorenz system (S3.3) with $(\sigma_1, \rho_1, \beta_1) = (10.010, 27.944, 2.667)$, $(\sigma_2, \rho_2, \beta_2) = (9.990, 28.056, 2.667)$, $\mu_{21} = 4$, and $\mu_{12} = 0$. Using the Euler scheme with step size 0.001 produces time series of duration 150, in which the data points from time instant 51 to 150 are used in the analysis. The observational functions are $u_i(x_i, y_i, z_i) = y_i$. The embedding parameters are $d_i = 7$ and $\tau_i = 2\omega$, where $i = 1, 2$, $\omega = 0.016$ is the sampling duration, $\boldsymbol{z} = \boldsymbol{u}_{1,2}$, and $E = 8$. It is clear that Condition [DD] holds from the trends in (a) and (b).

Therefore,

$$\begin{aligned}
\ln \|\boldsymbol{u}_{t+1} - \boldsymbol{u}^*\| &\approx \ln \left\| \tilde{\boldsymbol{f}}(\boldsymbol{u}_t, \boldsymbol{v}^*) - \boldsymbol{u}^* + \frac{\partial \tilde{\boldsymbol{f}}}{\partial \boldsymbol{v}}(\boldsymbol{u}_t, \boldsymbol{v}^*)(\boldsymbol{v}_t - \boldsymbol{v}^*) \right\| \\
&= \frac{1}{2} \ln \left\| \tilde{\boldsymbol{f}}(\boldsymbol{u}_t, \boldsymbol{v}^*) - \boldsymbol{u}^* + \frac{\partial \tilde{\boldsymbol{f}}}{\partial \boldsymbol{v}}(\boldsymbol{u}_t, \boldsymbol{v}^*)(\boldsymbol{v}_t - \boldsymbol{v}^*) \right\|^2 \\
&= \frac{1}{2} \ln \left\{ \left\| \tilde{\boldsymbol{f}}(\boldsymbol{u}_t, \boldsymbol{v}^*) - \boldsymbol{u}^* \right\|^2 + 2 \left[\tilde{\boldsymbol{f}}(\boldsymbol{u}_t, \boldsymbol{v}^*) - \boldsymbol{u}^* \right] \right. \\
&\quad \left. \cdot \left[\frac{\partial \tilde{\boldsymbol{f}}}{\partial \boldsymbol{v}}(\boldsymbol{u}_t, \boldsymbol{v}^*)(\boldsymbol{v}_t - \boldsymbol{v}^*) \right] + \left\| \frac{\partial \tilde{\boldsymbol{f}}}{\partial \boldsymbol{v}}(\boldsymbol{u}_t, \boldsymbol{v}^*)(\boldsymbol{v}_t - \boldsymbol{v}^*) \right\|^2 \right\} \\
&\approx \frac{1}{2} \ln \left\{ 1 + \frac{\left\| \tilde{\boldsymbol{f}}(\boldsymbol{u}_t, \boldsymbol{v}^*) - \boldsymbol{u}^* \right\| \left\| \frac{\partial \tilde{\boldsymbol{f}}}{\partial \boldsymbol{v}}(\boldsymbol{u}_t, \boldsymbol{v}^*)(\boldsymbol{v}_t - \boldsymbol{v}^*) \right\| \gamma_t}{\frac{1}{2} \left\| \tilde{\boldsymbol{f}}(\boldsymbol{u}_t, \boldsymbol{v}^*) - \boldsymbol{u}^* \right\|^2} \right\} \\
&\quad + \frac{1}{2} \ln \left(\left\| \tilde{\boldsymbol{f}}(\boldsymbol{u}_t, \boldsymbol{v}^*) - \boldsymbol{u}^* \right\|^2 \right) \\
&\approx \frac{\left\| \tilde{\boldsymbol{f}}(\boldsymbol{u}_t, \boldsymbol{v}^*) - \boldsymbol{u}^* \right\| \cdot \left\| \frac{\partial \tilde{\boldsymbol{f}}}{\partial \boldsymbol{v}}(\boldsymbol{u}_t, \boldsymbol{v}^*)(\boldsymbol{v}_t - \boldsymbol{v}^*) \right\| \cdot \gamma_t}{\left\| \tilde{\boldsymbol{f}}(\boldsymbol{u}_t, \boldsymbol{v}^*) - \boldsymbol{u}^* \right\|^2} \\
&\quad + \frac{1}{2} \ln \left(\left\| \tilde{\boldsymbol{f}}(\boldsymbol{u}_t, \boldsymbol{v}^*) - \boldsymbol{u}^* \right\|^2 \right) \\
&\approx \frac{\left\| \tilde{\boldsymbol{f}}(\boldsymbol{u}_t, \boldsymbol{v}^*) - \boldsymbol{u}^* \right\| \cdot \left\| \frac{\partial \tilde{\boldsymbol{f}}}{\partial \boldsymbol{v}}(\boldsymbol{u}_t, \boldsymbol{v}^*) \right\| \cdot \gamma_t}{\left\| \tilde{\boldsymbol{f}}(\boldsymbol{u}_t, \boldsymbol{v}^*) - \boldsymbol{u}^* \right\|^2} \|\boldsymbol{v}_t - \boldsymbol{v}^*\| \\
&\quad + \ln \left\| \tilde{\boldsymbol{f}}(\boldsymbol{u}_t, \boldsymbol{v}^*) - \boldsymbol{u}^* \right\|, \qquad \text{(S2.15)}
\end{aligned}$$

where γ_t denotes the cosine of the angle between vectors $\tilde{\boldsymbol{f}}(\boldsymbol{u}_t, \boldsymbol{v}^*) - \boldsymbol{u}^*$ and $\frac{\partial \tilde{\boldsymbol{f}}}{\partial \boldsymbol{v}}(\boldsymbol{u}_t, \boldsymbol{v}^*)(\boldsymbol{v}_t - \boldsymbol{v}^*)$. By replacing $\|\boldsymbol{u}_{t+1} - \boldsymbol{u}^*\|$ with $\varepsilon_{\boldsymbol{u}}$ and $\|\boldsymbol{v}_t - \boldsymbol{v}^*\|$ with $\delta^t_{\boldsymbol{v}}$, we obtain

$$\ln(\varepsilon_{\boldsymbol{u}}) \approx \tilde{K}^t_{\boldsymbol{v} \hookrightarrow \boldsymbol{u}} \delta^t_{\boldsymbol{v}} + \tilde{B}^t_{\boldsymbol{v} \hookrightarrow \boldsymbol{u}} \qquad \text{(S2.16)}$$

with the parameters abbreviated as $\tilde{K}^t_{\boldsymbol{v} \hookrightarrow \boldsymbol{u}}$ and $\tilde{B}^t_{\boldsymbol{v} \hookrightarrow \boldsymbol{u}}$. Because $\varepsilon_{\boldsymbol{u}}$ is independent of time t, reformulating this equation (subtract $\tilde{B}^t_{\boldsymbol{v} \hookrightarrow \boldsymbol{u}}$ and then divided by $\tilde{K}^t_{\boldsymbol{v} \hookrightarrow \boldsymbol{u}}$ at both sides of (S2.16)) and taking average over time admit

$$\langle \delta^t_{\boldsymbol{v}} \rangle_t \approx \langle K^t_{\boldsymbol{v} \hookrightarrow \boldsymbol{u}} \rangle_t \ln(\varepsilon_{\boldsymbol{u}}) + \langle B^t_{\boldsymbol{v} \hookrightarrow \boldsymbol{u}} \rangle_t, \qquad \text{(S2.17)}$$

which clearly demonstrates the linear scaling relation. Notice that this scaling relation can be further rewritten by substituting the average over time by the average over space based on ergodic theory, i.e.,

$$\int \delta_{\boldsymbol{v}} \, \mathrm{d}\mu_{\mathcal{L}_u \times \mathcal{L}_v} \approx \int K_{\boldsymbol{v} \hookrightarrow \boldsymbol{u}} \, \mathrm{d}\mu_{\mathcal{L}_u \times \mathcal{L}_v} \ln(\varepsilon_{\boldsymbol{u}}) + \int B_{\boldsymbol{v} \hookrightarrow \boldsymbol{u}} \, \mathrm{d}\mu_{\mathcal{L}_u \times \mathcal{L}_v}, \qquad \text{(S2.18)}$$

where $\mu_{\mathcal{L}_u \times \mathcal{L}_v}$ represents the Sinai-Ruelle-Bowen (SRB) measure [1] on $\mathcal{L}_u \times \mathcal{L}_v$.

III. ADDITIONAL EXAMPLES DEMONSTRATING THE POWER OF CONTINUITY SCALING FRAMEWORK IN ASCERTAINING AND QUANTIFYING CAUSATION

A. Unidirectionally and bidirectionally coupled systems

1. *Coupled ecological models*

The coupled map system is written as

$$x_{i,t+1} = x_{i,t}\left(r_i - r_i x_{i,t} - \sum_{j\neq i}\mu_{ij}x_{j,t}\right), \tag{S3.1}$$

where $i = 1,\ldots,N_V$, $t \in \mathbb{N}$. We consider two-species models here: $N_V = 2$ with $(r_1, r_2) = (3.8, 3.7)$ and choose different pairs of the coupling parameters $\{\mu_{ij}\}$. Time series of 5400 points are generated with the first 400 points abandoned to get rid of the transient behavior. The observational functions are $u_i(x_i) = x_i$ and the embedding parameters are $d_i = 3$ and $\tau_i = 1$ for $i = 1, 2$, and $E = 0$. In order to detect the causal relation $x_1 \hookrightarrow x_2$, we calculate the scaling relation between $\langle\delta^t_{\boldsymbol{u}_j}(\varepsilon_{\boldsymbol{u}_i})\rangle_{t\in\mathbb{N}}$ and $\ln\varepsilon_{\boldsymbol{u}_i}$ for $i, j = 1, 2$ with $i \neq j$, where $\boldsymbol{u_i}$ is the reconstructed state space vector from the observational time series $\{u_i(x_{i,t})\}_{t\in\mathbb{N}}$. In each case, the p-value is calculated with 25 random surrogates.

Case a: For the unidirectionally coupled two-species model, the scaling relations for different pairs of the coupling parameters have been shown in Figs. 3(a)-3(b) in the main text. The calculated slopes together with their p-values are listed in Tab. S2. The results validate our continuity scaling framework. Moreover, statistical fluctuation tests are performed with parameters $\mu_{12} = 0, \mu_{21} = 0.1$ and 400 uniformly generated grid initial values from $[0, 1] \times [0, 1]$. The CS results are presented in Fig. S2, showing high statistical robustness.

TABLE S2. Estimated slopes of the regression lines of the scaling relations and their p-values for the unidirectionally coupled ecological models.

No.	$s_{\boldsymbol{u}_1\hookrightarrow\boldsymbol{u}_2}$	$s_{\boldsymbol{u}_2\hookrightarrow\boldsymbol{u}_1}$	μ_{21}	μ_{12}	p-value ($s_{\boldsymbol{u}_1\hookrightarrow\boldsymbol{u}_2}$)	p-value ($s_{\boldsymbol{u}_2\hookrightarrow\boldsymbol{u}_1}$)
1	0.0004	0.0005	0.00	0.00	0.2680	0.1641
2	0.1167	-0.0002	0.05	0.00	0.0000	0.8385
3	0.1203	0.0006	0.10	0.00	0.0000	0.7673
4	0.1238	0.0005	0.15	0.00	0.0000	0.3864

Case b: For the bidirectionally coupled two-species model, the scaling relations for different parameter combinations are shown in Fig. S3. The slopes with their p-values are listed in Tab. S3, also providing validation to the continuity scaling framework. Statistical fluctuation tests showing high robustness of the CS framework are shown in Fig. S4.

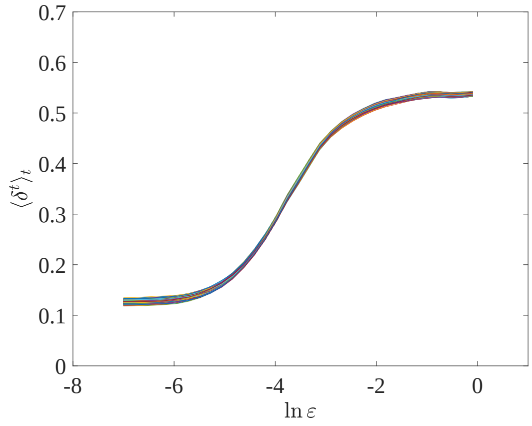

(a)

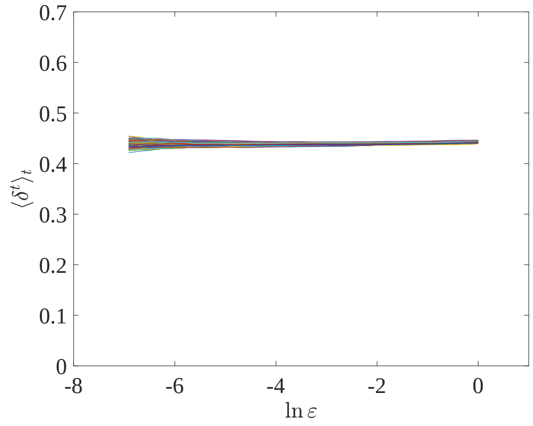

(b)

FIG. S2. Statistical fluctuation tests with the unidirectionally coupled two-species models (S3.1). Results for $\mu_{12} = 0$ and $\mu_{21} = 0.1$ and 400 grid initial values from $[0, 1] \times [0, 1]$ are shown. (a) The scaling relation for detecting the causation $x_1 \hookrightarrow x_2$ with mean CS index value 0.1240 and standard deviation 0.0015. (b) The scaling relation for detecting the causation $x_2 \hookrightarrow x_1$ with mean CS index value 0.0006 and standard deviation 0.0006.

Case c: For the unidirectionally coupled two-species model subject to noise:

$$x_{i,t+1} = x_{i,t}\left(r_i - r_i x_{i,t} - \sum_{j\neq i} \mu_{ij} x_{j,t}\right) + U_{i,t}, \quad i = 1, 2, \tag{S3.2}$$

where $\{U_{1,t}, U_{2,t}\}_{\mathbb{N}}$ are i.i.d. random variables uniformly distributed on $[-\sigma, \sigma]$ and $\mu_{21} = 0.1$, the representative scaling relations are shown in Fig. S5 and the estimated slopes with their p-values are listed in Tab. S4, demonstrating good robustness of our framework against noise.

TABLE S3. Estimated slopes of the regression lines associated with the scaling relations in Fig. S3 and their p-values for the bidirectionally coupled two-species model.

No.	$s_{\boldsymbol{u}_1 \hookrightarrow \boldsymbol{u}_2}$	$s_{\boldsymbol{u}_2 \hookrightarrow \boldsymbol{u}_1}$	μ_{21}	μ_{12}	p-value ($s_{\boldsymbol{u}_1 \hookrightarrow \boldsymbol{u}_2}$)	p-value ($s_{\boldsymbol{u}_2 \hookrightarrow \boldsymbol{u}_1}$)
1	0.0007	0.0929	0.00	0.05	0.1555	0.0000
2	0.1092	0.0986	0.05	0.05	0.0000	0.0000
3	0.1183	0.0976	0.10	0.05	0.0000	0.0000
4	0.1217	0.0937	0.15	0.05	0.0000	0.0000

2. *Coupled Lorenz systems*

The system equations are

$$\begin{aligned} \dot{x}_{i,t} &= \sigma_i(y_{i,t} - x_{i,t}) + \textstyle\sum_{j\neq i} \mu_{ij} x_{j,t}, \\ \dot{y}_{i,t} &= x_{i,t}(\rho_i - z_{i,t}) - y_{i,t}, \\ \dot{z}_{i,t} &= x_{i,t} y_{i,t} - \beta_i z_{i,t}, \end{aligned} \tag{S3.3}$$

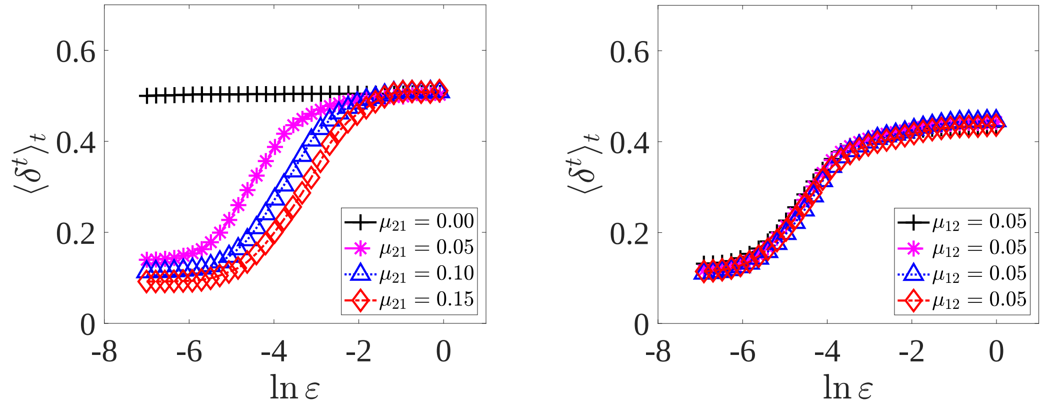

(a) (b)

FIG. S3. Detecting causation in the bidirectionally coupled two-species models (S3.1). Results for fixed $\mu_{12} = 0.05$ and a number of μ_{21} values are shown. (a) The scaling relation between $\langle\delta^t_{\boldsymbol{u}_1}(\varepsilon_{\boldsymbol{u}_2})\rangle_t$ and $\ln\varepsilon_{\boldsymbol{u}_2}$ for detecting the causation $x_1 \hookrightarrow x_2$. (b) The scaling relation between $\langle\delta^t_{\boldsymbol{u}_2}(\varepsilon_{\boldsymbol{u}_1})\rangle_t$ and $\ln\varepsilon_{\boldsymbol{u}_1}$ for detecting the causation $x_2 \hookrightarrow x_1$.

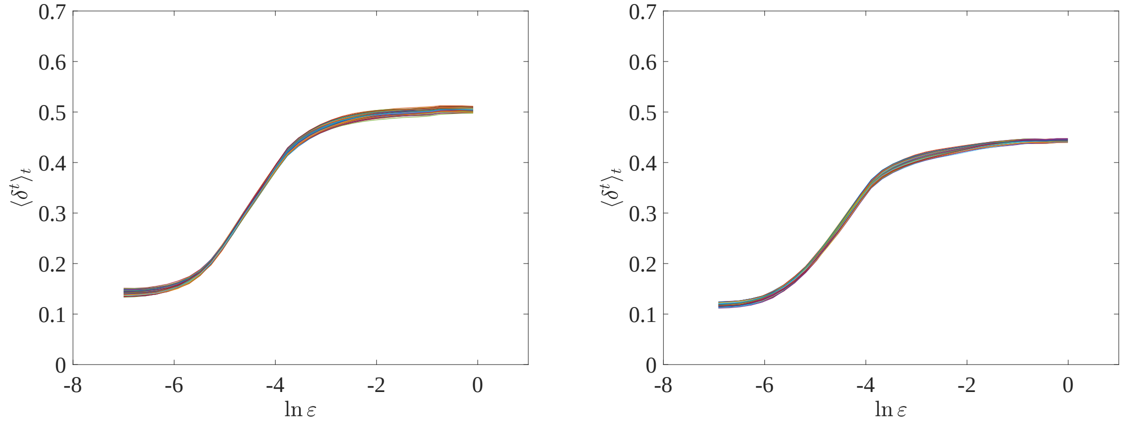

(a) (b)

FIG. S4. Statistical fluctuation tests with the bidirectionally coupled two-species models (S3.1). Results for $\mu_{12} = 0.05$ and $\mu_{21} = 0.05$ and 400 grid initial values from $[0,1] \times [0,1]$ are shown. (a) The scaling relation for detecting the causation $x_1 \hookrightarrow x_2$ with mean CS index value 0.1123 and standard deviation 0.0016. (b) The scaling relation for detecting the causation $x_2 \hookrightarrow x_1$ with mean CS index value 0.0975 and standard deviation 0.0014.

where $i = 1, \ldots, N_V,\ t \in \mathbb{R}$. Here we set $N_V = 2$, $(\sigma_1, \rho_1, \beta_1) = (10.010, 27.944, 2.667)$, and $(\sigma_2, \rho_2, \beta_2) = (9.990, 28.056, 2.667)$. Different combinations of the coupling parameters are used to validate the continuity scaling framework for this continuous-time dynamical system. Euler scheme is used with step size 0.001 to produce time series of duration 150 and the data points from time instant 51 to 150 are used in the analysis. The observational functions are $u_i(x_i, y_i, z_i) = y_i$. The embedding parameters are $d_i = 7$ and $\tau_i = 2\omega$, where $i = 1, 2$. Sampling duration $\omega = 0.016$, and $E = 8$. Each p-value is calculated with 20 random surrogates.

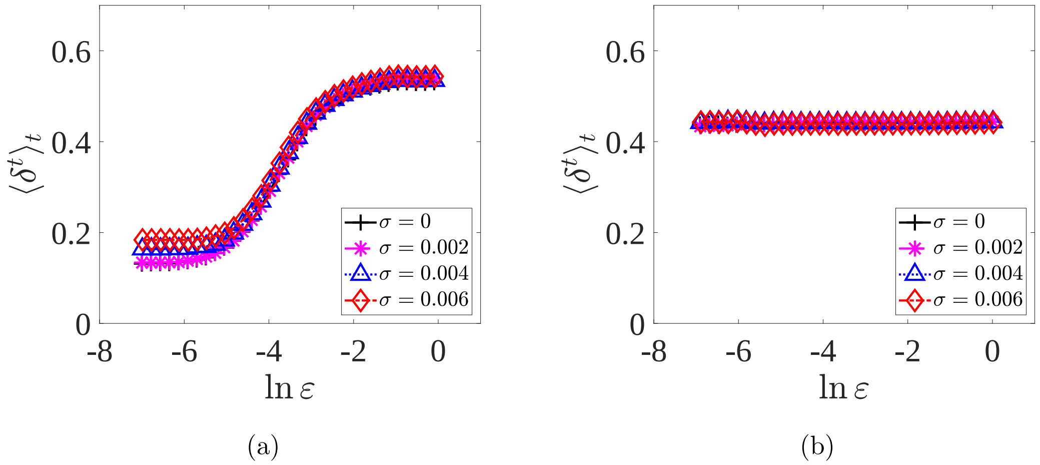

FIG. S5. Detecting causation in the unidirectionally coupled two-species system (S3.2) under noise perturbation. The fixed parameters are $\mu_{12} = 0$ and $\mu_{21} = 0.1$. The noise amplitude σ varies as indicated inside each panel. (a) The scaling relation between $\langle \delta^t_{\boldsymbol{u}_1}(\varepsilon_{\boldsymbol{u}_2}) \rangle_t$ and $\ln \varepsilon_{\boldsymbol{u}_2}$ for detecting the causal relation $x_1 \hookrightarrow x_2$. (b) The scaling relation between $\langle \delta^t_{\boldsymbol{u}_2}(\varepsilon_{\boldsymbol{u}_1}) \rangle_t$ and $\ln \varepsilon_{\boldsymbol{u}_1}$ nullifying the causal relation $x_2 \hookrightarrow x_1$.

TABLE S4. Estimated slopes of the regression lines associated with the scaling relations in Fig. S5 and their p-values for the unidirectionally coupled two-species model for different values of the noise amplitude.

No.	$s_{\boldsymbol{u}_1 \hookrightarrow \boldsymbol{u}_2}$	$s_{\boldsymbol{u}_2 \hookrightarrow \boldsymbol{u}_1}$	σ	p-value ($s_{\boldsymbol{u}_1 \hookrightarrow \boldsymbol{u}_2}$)	p-value ($s_{\boldsymbol{u}_2 \hookrightarrow \boldsymbol{u}_1}$)
1	0.1203	0.0006	0.000	0.0000	0.1637
2	0.1203	0.0011	0.002	0.0000	0.9373
3	0.1149	-0.0000	0.004	0.0000	0.5167
4	0.1131	-0.0001	0.006	0.0000	0.2990

Case d: For the unidirectionally coupled Lorenz system, the continuity scaling relations are shown in Fig. S6 with the slopes and their p-values listed in Tab. S5. Moreover, statistical fluctuation tests are performed with parameters $\mu_{12} = 0, \mu_{21} = 3$ and 400 randomly generated initial values from $[-3, 3] \times [-3, 3] \times [-3, 3]$. The CS results are presented in Fig. S7, showing high statistical robustness.

Case e: For the bidirectionally coupled Lorenz system, the scaling relations are shown in Fig. S8 with the slopes and their p-values listed in Tab. S6. Statistical fluctuation tests showing high robustness of the CS framework are shown in Fig. S9.

Case f: The unidirectionally coupled Lorenz system with noise perturbation is given by

$$\begin{aligned}
\mathrm{d}x_{i,t} &= \left(\sigma_i(y_{i,t}-x_{i,t})+\textstyle\sum_{j\neq i}\mu_{ij}x_{j,t}\right)\mathrm{d}t+\sigma\cdot\mathrm{d}W_{i,x,t},\\
\mathrm{d}y_{i,t} &= (x_{i,t}(\rho_i-z_{i,t})-y_{i,t})\mathrm{d}t+\sigma\cdot\mathrm{d}W_{i,y,t},\\
\mathrm{d}z_{i,t} &= (x_{i,t}y_{i,t}-\beta_i z_{i,t})\mathrm{d}t+\sigma\cdot\mathrm{d}W_{i,z,t},
\end{aligned} \tag{S3.4}$$

where $i=1,2,\ t\in\mathbb{R}$ and each $W_i=\{(W_{i,x,t},W_{i,y,t},W_{i,z,t})\}_t$ is a standard Wiener process of dimension three, $W_{1,2}$ are mutually independent, $\mu_{21}=4$, and σ is the noise amplitude. The scaling relations are shown in Fig. S10 with the slopes and their p-values listed in Tab. S7. Results demonstrate good robustness against noise for our continuity scaling framework.

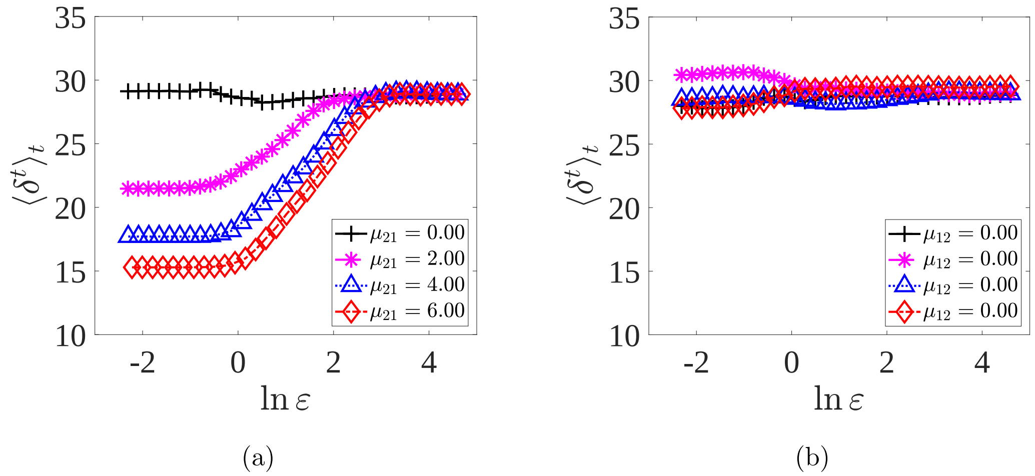

FIG. S6. Causal detection in the unidirectionally coupled Lorenz system (S3.3). Various values of μ_{21} are indicated inside the left panel. (a) Scaling relation between $\langle\delta^t_{\boldsymbol{u}_1}(\varepsilon_{\boldsymbol{u}_2})\rangle_t$ and $\ln\varepsilon_{\boldsymbol{u}_2}$ for identifying the causal relation $x_1\hookrightarrow x_2$. (b) Scaling relation between $\langle\delta^t_{\boldsymbol{u}_2}(\varepsilon_{\boldsymbol{u}_1})\rangle_t$ and $\ln\varepsilon_{\boldsymbol{u}_1}$ for demonstrating nonexistence of the causal relation $x_2\hookrightarrow x_1$.

TABLE S5. Slopes of the regression lines associated with the scaling relations in Fig. S6 and their p-values for the unidirectionally coupled Lorenz system.

No.	$s_{\boldsymbol{u}_1\hookrightarrow\boldsymbol{u}_2}$	$s_{\boldsymbol{u}_2\hookrightarrow\boldsymbol{u}_1}$	μ_{21}	μ_{12}	p-value ($s_{\boldsymbol{u}_1\hookrightarrow\boldsymbol{u}_2}$)	p-value ($s_{\boldsymbol{u}_2\hookrightarrow\boldsymbol{u}_1}$)
1	-0.0620	0.1363	0.00	0.00	0.7247	0.2292
2	2.4251	-0.2906	2.00	0.00	0.0000	0.9237
3	3.5427	0.0279	4.00	0.00	0.0000	0.6066
4	4.3265	0.2967	6.00	0.00	0.0000	0.1916

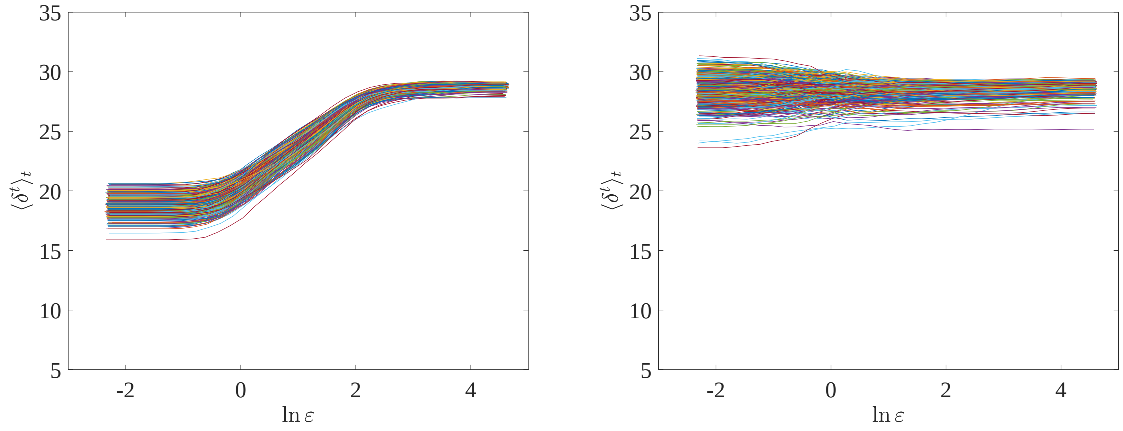

(a) (b)

FIG. S7. Statistical fluctuation tests with the unidirectionally coupled Lorenz system (S3.3). Results for $\mu_{12} = 0$ and $\mu_{21} = 3$ and 400 randomly generated initial values from $[-3, 3] \times [-3, 3] \times [-3, 3]$ are shown. (a) The scaling relation for detecting the causation $x_1 \hookrightarrow x_2$ with mean CS index value 3.1231 and standard deviation 0.2538. (b) The scaling relation for detecting the causation $x_2 \hookrightarrow x_1$ with mean CS index value 0.0575 and standard deviation 0.2004.

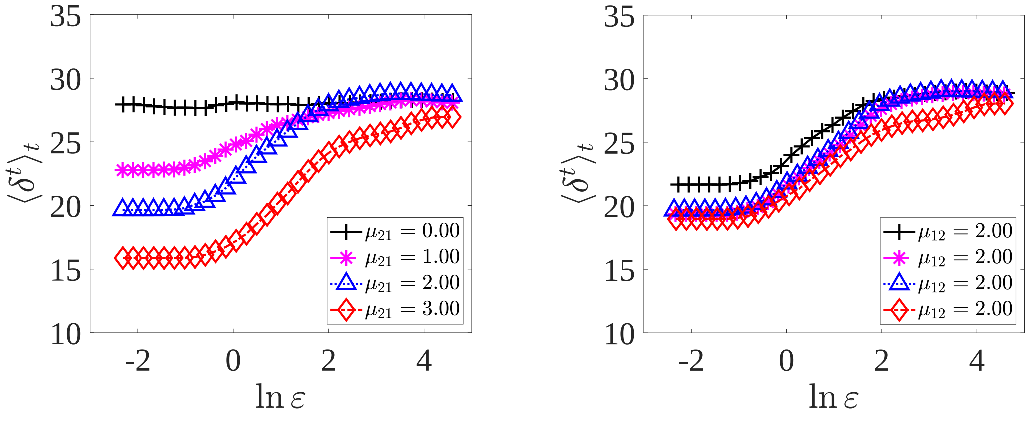

(a) (b)

FIG. S8. Detecting causation in the bidirectionally coupled Lorenz system (S3.3) for fixed $\mu_{12} = 2$. Various values of μ_{21} are indicated inside the left panel. (a) Scaling relation between $\langle \delta^t_{\boldsymbol{u}_1}(\varepsilon_{\boldsymbol{u}_2}) \rangle_t$ and $\ln \varepsilon_{\boldsymbol{u}_2}$ for ascertaining the causal relation $x_1 \hookrightarrow x_2$. (b) Scaling relation between $\langle \delta^t_{\boldsymbol{u}_2}(\varepsilon_{\boldsymbol{u}_1}) \rangle_t$ and $\ln \varepsilon_{\boldsymbol{u}_1}$ for confirming the causal relation $x_2 \hookrightarrow x_1$.

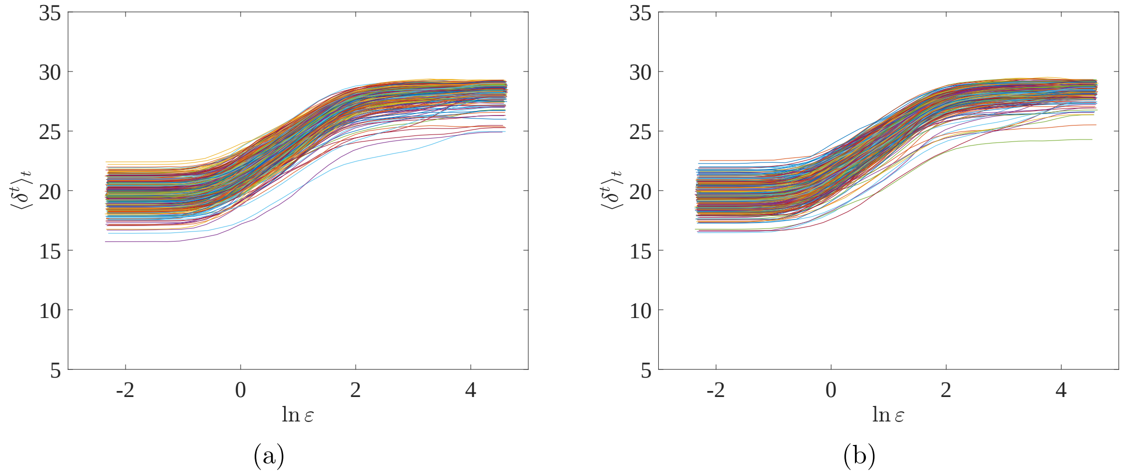

FIG. S9. Statistical fluctuation tests with the bidirectionally coupled Lorenz system (S3.3). Results for $\mu_{12} = 2$ and $\mu_{21} = 2$ and 400 randomly generated initial values from $[-3,3] \times [-3,3] \times [-3,3]$ are shown. (a) The scaling relation for detecting the causation $x_1 \hookrightarrow x_2$ with mean CS index value 2.5565 and standard deviation 0.4125. (b) The scaling relation for detecting the causation $x_2 \hookrightarrow x_1$ with mean CS index value 2.5861 and standard deviation 0.4073.

TABLE S6. Slopes of the regression lines associated with the scaling relations in Fig. S8 and their p-values for the bidirectionally coupled Lorenz system.

No.	$s_{\boldsymbol{u}_1 \hookrightarrow \boldsymbol{u}_2}$	$s_{\boldsymbol{u}_2 \hookrightarrow \boldsymbol{u}_1}$	μ_{21}	μ_{12}	p-value ($s_{\boldsymbol{u}_1 \hookrightarrow \boldsymbol{u}_2}$)	p-value ($s_{\boldsymbol{u}_2 \hookrightarrow \boldsymbol{u}_1}$)
1	0.0695	2.2773	0.00	2.00	0.3754	0.0001
2	1.2333	2.8261	1.00	2.00	0.0000	0.0000
3	2.8006	2.8561	2.00	2.00	0.0000	0.0000
4	2.7493	1.9162	3.00	2.00	0.0000	0.0002

TABLE S7. Slopes of the regression lines associated with the scaling relations in Fig. S10 and their p-values for the unidirectionally coupled Lorenz system under noise perturbation of different amplitudes.

No.	$s_{\boldsymbol{u}_1 \hookrightarrow \boldsymbol{u}_2}$	$s_{\boldsymbol{u}_2 \hookrightarrow \boldsymbol{u}_1}$	σ	p-value ($s_{\boldsymbol{u}_1 \hookrightarrow \boldsymbol{u}_2}$)	p-value ($s_{\boldsymbol{u}_2 \hookrightarrow \boldsymbol{u}_1}$)
1	3.5427	0.0279	0.00	0.0000	0.6275
2	3.2192	-0.2594	0.08	0.0000	0.7441
3	3.4558	0.1312	0.16	0.0000	0.4412
4	3.1478	0.1133	0.24	0.0000	0.5063

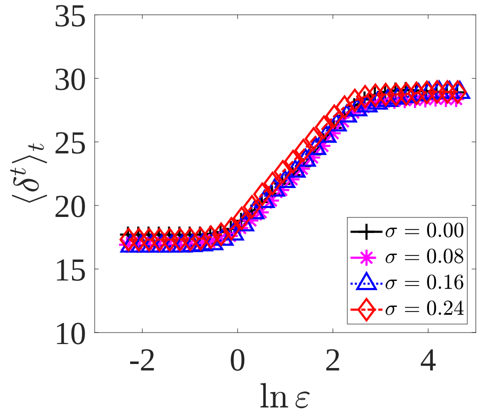

(a)

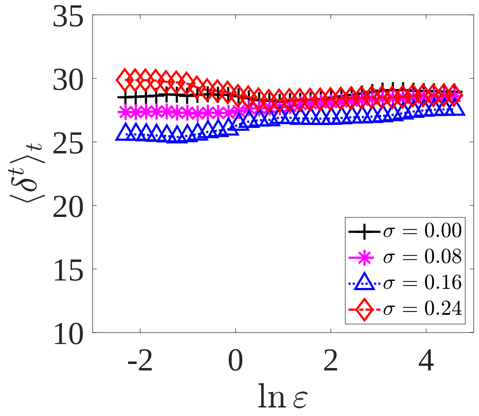

(b)

FIG. S10. Detecting causation in the unidirectionally coupled Lorenz system (S3.4) under noise perturbations. The coupling parameter values are $\mu_{12} = 0$ and $\mu_{21} = 4$. The various values of the noise amplitude σ are indicated inside each panel. (a) Scaling relation between $\langle \delta^t_{\boldsymbol{u}_1}(\varepsilon_{\boldsymbol{u}_2}) \rangle_t$ and $\ln \varepsilon_{\boldsymbol{u}_2}$ for ascertaining the causal relation $x_1 \hookrightarrow x_2$. (b) Scaling relation between $\langle \delta^t_{\boldsymbol{u}_2}(\varepsilon_{\boldsymbol{u}_1}) \rangle_t$ and $\ln \varepsilon_{\boldsymbol{u}_1}$ demonstrating nonexistence of the causal relation $x_2 \hookrightarrow x_1$.

3. Coupled Rössler-Lorenz systems

The system equations are

$$
\begin{aligned}
\dot{x}_{1,t} &= -\alpha(y_{1,t} + z_{1,t}), \\
\dot{y}_{1,t} &= \alpha(x_{1,t} + 0.2 y_{1,t}), \\
\dot{z}_{1,t} &= \alpha[0.2 + z_{1,t}(x_{1,t} - 5.7)], \\
\\
\dot{x}_{2,t} &= \sigma(y_{2,t} - z_{2,t}) \ + \mu_{21} y_{1,t}, \\
\dot{y}_{2,t} &= x_{2,t}(\rho - z_{2,t}) - y_{2,t}, \\
\dot{z}_{2,t} &= x_{2,t} y_{2,t} - \beta z_{2,t},
\end{aligned}
\tag{S3.5}
$$

where $\alpha = 6$, $\sigma = 10.01$, $\rho = 28.028$, and $\beta = 2.664$. There is unidirectional coupling from the Rössler to the Lorenz system. Time series of duration 280 are generated using the Euler scheme with the step size 0.001. The data points before $t = 81$ are disregarded to eliminate transient behaviors. The observational functions are $u_1(x_1, y_1, z_1) = x_1 + 0.01 y_1 + 0.1 \sin(z_1)$ and $u_2(x_2, y_2, z_2) = y_2 - 0.01 x_2 + 0.1 \cos(z_2)$. Other parameters are: sampling duration $\omega = 0.016$, embedding dimensions $d_1 = 13$ and $d_2 = 8$, time delay $\tau_1 = 2\omega$ and $\tau_2 = 3\omega$, $E = 10$, and 20 random surrogates are used to calculate the p-value. The scaling relations are displayed in Fig. S11 and the corresponding slopes together with their p-values are listed in Tab. S8. All the results show our continuity scaling framework is highly effective in detecting causation in nonlinear dynamical systems.

B. Effects of varying sampling duration

To assess the effects of sampling duration on causation detection in continuous-time dynamical systems, we take the unidirectionally coupled Lorenz system (S3.3) with $N_V = 2$

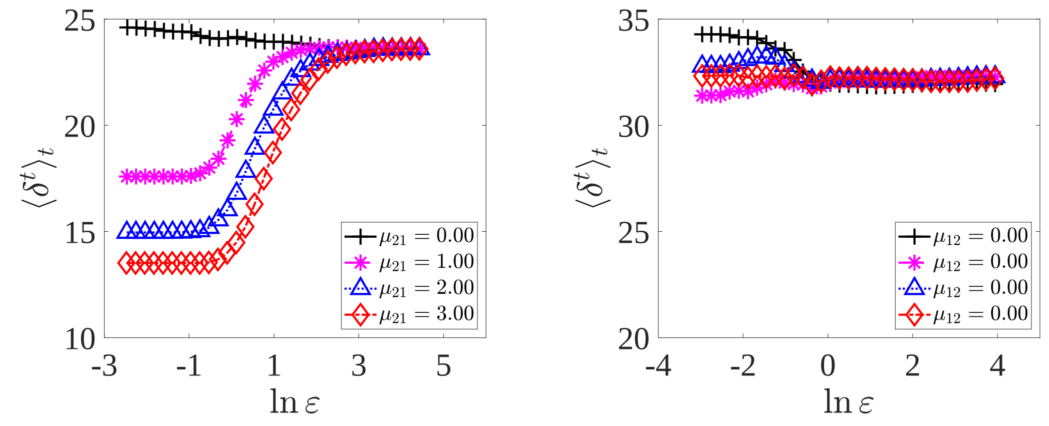

(a) (b)

FIG. S11. Detecting causation in the unidirectionally coupled Rössler-Lorenz system (S3.5). The value of the coupling parameter μ_{21} is varied systematically from $\{0, 1, 2, 3\}$, while the parameter μ_{12} is set to be zero (unidirectional coupling from the Rössler to the Lorenz system). (a) Scaling relation between $\langle\delta^t_{\boldsymbol{u}_1}(\varepsilon_{\boldsymbol{u}_2})\rangle_t$ and $\ln\varepsilon_{\boldsymbol{u}_2}$ for ascertaining the causal relation $(x_1, y_1, z_1) \hookrightarrow (x_2, y_2, z_2)$. (b) Scaling relation between $\langle\delta^t_{\boldsymbol{u}_2}(\varepsilon_{\boldsymbol{u}_1})\rangle_t$ and $\ln\varepsilon_{\boldsymbol{u}_1}$ nullifying the causal relation $(x_2, y_2, z_2) \hookrightarrow (x_1, y_1, z_1)$.

TABLE S8. Slopes of the regression lines associated with the scaling relations in Fig. S11 and their p-values for the unidirectionally coupled Rössler-Lorenz system.

No.	$s_{\boldsymbol{u}_1\hookrightarrow\boldsymbol{u}_2}$	$s_{\boldsymbol{u}_2\hookrightarrow\boldsymbol{u}_1}$	μ_{21}	μ_{12}	p-value ($s_{\boldsymbol{u}_1\hookrightarrow\boldsymbol{u}_2}$)	p-value ($s_{\boldsymbol{u}_2\hookrightarrow\boldsymbol{u}_1}$)
1	-0.1596	-0.3893	0.00	0.00	0.8617	0.9756
2	2.1836	0.1440	1.00	0.00	0.0000	0.2468
3	2.8633	-0.1323	2.00	0.00	0.0000	0.8563
4	3.4286	-0.0322	3.00	0.00	0.0000	0.7518

as an example. The simulation setting in terms of the system parameters, the observational functions, and the embedding dimension, is the same as that in section III.A.2. We conduct 60 numerical runs: for $\mu_{21} = \{0, 2, 4, 6\}$ and $\{\omega^k\}_{k=1,\cdots,15}$. The delay time $\tau = \tau^k$, the threshold $E = E^k$, and the length of the time series $L = 50 + T^k$ are used and shown in Tab. S9. The calculated slope values of the continuity scaling are shown in Fig. 4, with more details listed in Tab. S9. Notice that even with relatively low sampling rate, our continuity scaling framework can successfully detect and quantify the strength of causation.

C. Additional examples with complex nonlinear coupling schemes

In addition to the linear coupling schemes as we discussed for the coupled Lorenz system (S3.3), our continuity scaling framework is also effective for nonlinear couplings as in the coupled ecological system (S3.1). To further investigate the universality of

TABLE S9. Slopes of the regression lines associated with the continuity scaling for the unidirectionally coupled Lorenz system with different sampling durations.

	$s_{\boldsymbol{u}_1 \hookrightarrow \boldsymbol{u}_2}$				$s_{\boldsymbol{u}_2 \hookrightarrow \boldsymbol{u}_1}$							
	μ_{21}	μ_{21}	μ_{21}	μ_{21}	μ_{12}	μ_{12}	μ_{12}	μ_{12}				
k	0	2	4	6	0	0	0	0	ω^k	τ^k	E^k	T^k
1	0.200	2.349	4.519	4.375	0.234	0.205	0.041	0.275	0.004	0.048	20	60
2	0.067	3.115	4.930	4.578	0.131	0.308	0.144	0.030	0.005	0.050	20	75
3	-0.210	3.118	4.573	4.574	0.100	0.348	0.200	0.002	0.006	0.048	16	80
4	-0.209	3.966	4.164	4.751	-0.101	0.273	0.082	0.143	0.007	0.049	16	90
5	0.016	3.004	4.545	4.830	-0.172	0.009	0.169	0.189	0.008	0.048	16	100
6	-0.045	3.377	4.573	5.290	-0.073	-0.018	0.251	0.169	0.009	0.054	12	100
7	-0.073	3.139	4.305	5.004	-0.083	0.102	0.215	-0.011	0.010	0.050	12	100
8	0.028	3.467	4.624	5.366	-0.077	-0.005	0.034	0.165	0.011	0.055	12	100
9	-0.077	2.864	4.153	4.853	-0.108	-0.017	0.094	-0.035	0.012	0.048	8	100
10	-0.071	3.208	4.445	5.176	-0.213	0.282	0.171	0.077	0.013	0.052	8	100
11	0.073	2.727	3.761	4.457	-0.301	-0.002	0.138	0.056	0.014	0.042	8	100
12	-0.209	2.751	3.895	4.697	-0.032	-0.005	0.153	0.094	0.015	0.045	8	100
13	0.122	2.858	4.116	4.899	0.118	-0.285	0.306	0.089	0.016	0.048	8	100
14	-0.104	3.165	4.334	5.126	-0.039	0.036	0.260	0.319	0.017	0.051	8	100
15	0.093	3.143	4.535	5.331	0.200	0.020	0.172	0.223	0.018	0.054	8	100

continuity scaling in systems with more complex nonlinear coupling schemes, we consider the following examples. For the first example, we use the unidirectionally coupled Lorenz systems:

$$
\begin{aligned}
\dot{x}_{1,t} &= \sigma_1(y_{1,t} - x_{1,t}),\\
\dot{y}_{1,t} &= x_{1,t}(\rho_1 - z_{1,t}) - y_{1,t},\\
\dot{z}_{1,t} &= x_{1,t}y_{1,t} - \beta_1 z_{1,t},\\
\\
\dot{x}_{2,t} &= \sigma_2(y_{2,t} - x_{2,t}) + \mu_{21}\Omega(x_{1,t}, y_{1,t}, z_{1,t}),\\
\dot{y}_{2,t} &= x_{2,t}(\rho_2 - z_{2,t}) - y_{2,t},\\
\dot{z}_{2,t} &= x_{2,t}y_{2,t} - \beta_2 z_{2,t}
\end{aligned}
\tag{S3.6}
$$

with the nonlinear coupling function $\Omega(x, y, z) = x^3/400 + 20\sin(0.1x)$. System parameters are taken as: $(\sigma_1, \rho_1, \beta_1) = (10.010, 27.944, 2.667)$ and $(\sigma_2, \rho_2, \beta_2) = (9.990, 28.056, 2.667)$. Euler scheme with time step 0.001 is used to generate the time series with a length of 150, and the points in the first time duration, 50, are discarded to eliminate the transient states. We set the sampling duration as $\omega = 0.016$, the embedding dimension and the delay time as, respectively, 7ω and 2ω, and as $e = 0.001$, $N_\varepsilon = 33$, and $E = 8$. The observational functions are supposed to be $u_i(x_i, y_i, z_i) = y_i$, $i = 1, 2$. We change the coupling parameter μ_{21} in the set $\{0, 1, 2, 3\}$ and keep $\mu_{12} = 0$ (viz. unidirectional coupling). There are 20 surrogates generated for calculating the corresponding p-values. As shown in Fig. S12 and Tab. S10, the continuity scaling is still effective even with very complex nonlinear couplings, further demonstrating the universality of our framework.

As another illustrative example with nonlinear coupling, we use the unidirectionally

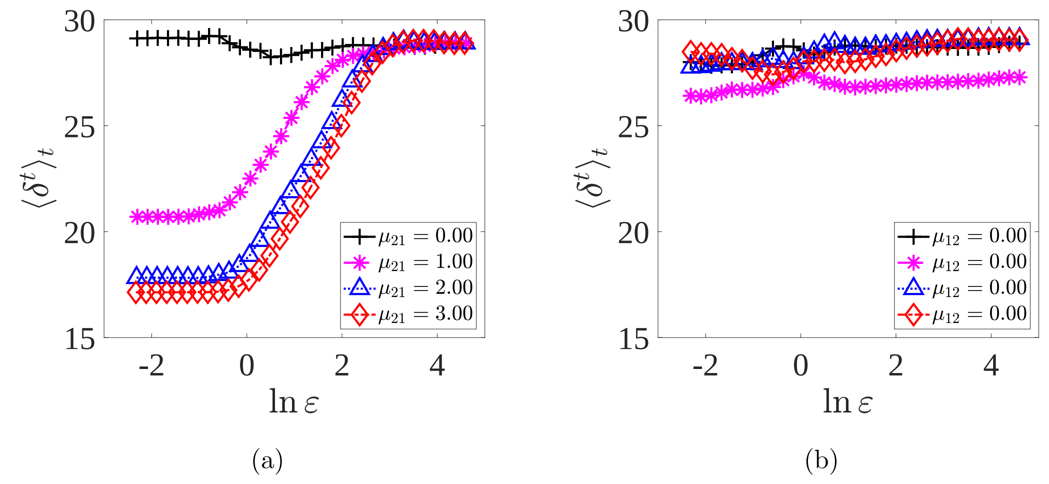

FIG. S12. Detecting causation in the unidirectionally nonlinearly coupled Lorenz system (S3.6). The value of the coupling parameter μ_{21} is varied systematically from $\{0, 1, 2, 3\}$, while the parameter μ_{12} is set to be zero. (a) Scaling relation between $\langle \delta^t_{\boldsymbol{u}_1}(\varepsilon_{\boldsymbol{u}_2}) \rangle_t$ and $\ln \varepsilon_{\boldsymbol{u}_2}$ for ascertaining the causal relation $x_1 \hookrightarrow x_2$. (b) Scaling relation between $\langle \delta^t_{\boldsymbol{u}_2}(\varepsilon_{\boldsymbol{u}_1}) \rangle_t$ and $\ln \varepsilon_{\boldsymbol{u}_1}$ nullifying the causal relation $x_2 \hookrightarrow x_1$.

TABLE S10. Slopes of the regression lines associated with the scaling relations in Fig. S12 and their p-values for the unidirectionally nonlinearly coupled Lorenz system.

No.	$s_{\boldsymbol{u}_1 \hookrightarrow \boldsymbol{u}_2}$	$s_{\boldsymbol{u}_2 \hookrightarrow \boldsymbol{u}_1}$	μ_{21}	μ_{12}	p-value ($s_{\boldsymbol{u}_1 \hookrightarrow \boldsymbol{u}_2}$)	p-value ($s_{\boldsymbol{u}_2 \hookrightarrow \boldsymbol{u}_1}$)
1	-0.06196	0.13625	0	0	0.6560	0.1497
2	2.28164	0.08594	1	0	0.0000	0.4996
3	3.47241	0.24354	2	0	0.0000	0.4427
4	3.77883	0.38925	3	0	0.0000	0.3290

coupled discrete-time Hénon system:

$$\begin{aligned} x_{1,t+1} &= 1 - a_1 x_{1,t}^2 + y_{1,t}, \\ y_{1,t+1} &= b_1 x_{1,t}, \\ \\ x_{2,t+1} &= 1 - a_2 x_{2,t}^2 + y_{2,t} + \mu_{21} \Omega(x_{1,t}, y_{1,t}), \\ y_{2,t+1} &= b_2 x_{2,t} \end{aligned} \tag{S3.7}$$

with the coupling function $\Omega(x, y) = \frac{2}{3} x^2 - \frac{1}{5} \sin(x)$. Time series of length 20400 are generated with the first 400 points that are discarded to eliminate the transient states. We set the sampling duration as $\omega = 4$, change the coupling strength μ_{21} in the set $\{0, 0.015, 0.030, 0.045\}$ and keep $\mu_{12} = 0$ (viz. unidirectional coupling). We set the system parameters as $[a_1, b_1] = [1.4, 0.2]$, $[a_2, b_2] = [1.401, 0.201]$ and the observational functions as $u_1(x_1, y_1) = x_1$ and $u_2(x_2, y_2) = x_2$. Additionally, we select the embedding dimension and the delay time as, respectively, 5ω and 1ω, and as $E = 0$, $e = 0.001$, and $N_\varepsilon = 33$.

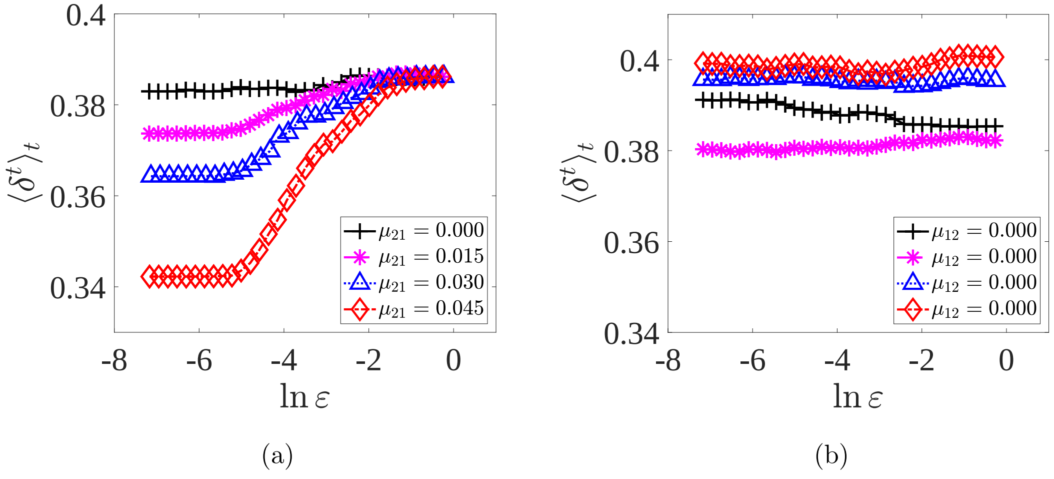

FIG. S13. Detecting causation in the unidirectionally nonlinearly coupled Hénon system (S3.7). The value of the coupling parameter μ_{21} is varied systematically from $\{0, 0.015, 0.030, 0.045\}$, while the parameter μ_{12} is set to be zero. (a) Scaling relation between $\langle \delta^t_{\boldsymbol{u}_1}(\varepsilon_{\boldsymbol{u}_2}) \rangle_t$ and $\ln \varepsilon_{\boldsymbol{u}_2}$ for ascertaining the causal relation $x_1 \hookrightarrow x_2$. (b) Scaling relation between $\langle \delta^t_{\boldsymbol{u}_2}(\varepsilon_{\boldsymbol{u}_1}) \rangle_t$ and $\ln \varepsilon_{\boldsymbol{u}_1}$ nullifying the causal relation $x_2 \hookrightarrow x_1$.

TABLE S11. Slopes of the regression lines associated with the scaling relations in Fig. S13 and their p-values for the unidirectionally nonlinearly coupled Hénon system.

No.	$s_{\boldsymbol{u}_1 \hookrightarrow \boldsymbol{u}_2}$	$s_{\boldsymbol{u}_2 \hookrightarrow \boldsymbol{u}_1}$	μ_{21}	μ_{12}	p-value ($s_{\boldsymbol{u}_1 \hookrightarrow \boldsymbol{u}_2}$)	p-value ($s_{\boldsymbol{u}_2 \hookrightarrow \boldsymbol{u}_1}$)
1	0.000556	-0.001033	0.000	0.00	0.1210	0.9876
2	0.003331	0.000539	0.015	0.00	0.0000	0.2447
3	0.005790	-0.000204	0.030	0.00	0.0000	0.6443
4	0.012026	0.000077	0.045	0.00	0.0000	0.5384

There are 28 surrogates generated to calculate the p-values. The results presented in Fig. S13 and Tab. S11 also validate the efficacy of our framework of the continuity scaling.

In addition, to show the superior efficacy of our framework to the other existing methods in detecting causation in the form of the nonlinear couplings, we present here a comparison on the unidirectionally coupled Hénon system (S3.7) with the continuity scaling framework and the CCM technique. We depict the index values for the continuity scaling (the slope) and the CCM (see section IV) as the coupling parameter μ_{21} increasing from 0 to 0.04 and μ_{12} sustaining at zero. All the other parameters are set in the same manner as those used in the above example. Figure S14 shows a clear distinction for the directions with and without causations for the continuity scaling. However, the CCM can hardly distinguish the correct causation [see the two intertwined curves in Fig. S14(b)], even bringing reversal, wrong identifications for some coupling strengths.

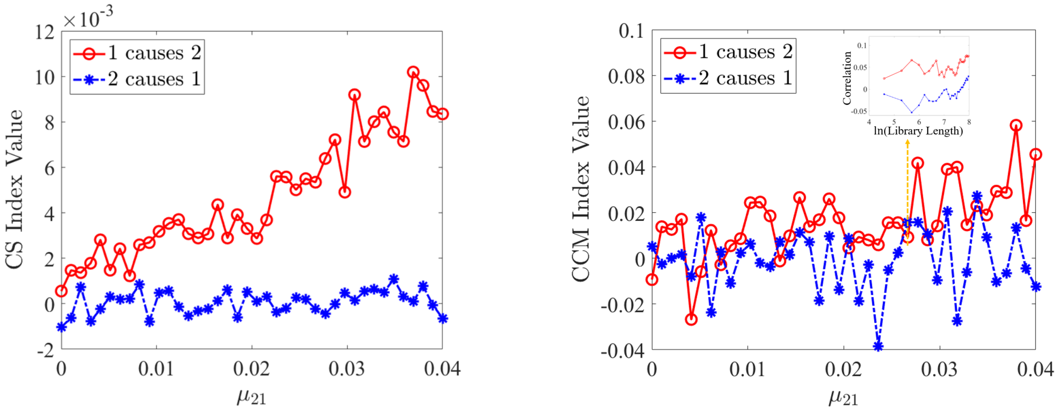

FIG. S14. Comparison of causation detection with continuity scaling and CCM for the unidirectionally nonlinearly coupled Hénon system (S3.7). For CCM, the embedding dimension and delay time are 5ω and 1ω respectively and the length of library time series varies from 100 to 2900. Notice that CCM has reversed wrong identifications for some coupling strengths (see the subpanel for the corresponding converging curves [2]).

D. Inferring networks of causal interactions

We test the power of our continuity scaling framework in inferring networks of causal interactions from multivariate time series. Here the pairwise inference is considered while generalization of the CS framework to multivariate version will be included in our future work. To be concrete, each network has 5 nodes (i.e., $N_v = 5$) that interact with each other according to the chain, ring, or tree topology, as shown in Figs. S15(a)-15(c).

Discrete time nodal dynamics. We consider the case where the nodal dynamical system is the ecological model (S3.1). For convenience, we rewrite the system equation here:

$$x_{i,t+1} = x_{i,t}\left(r_i - r_i x_{i,t} - \sum_{j\neq i} \mu_{ij} x_{j,t}\right),$$

where $i = 1, \dots, N_V,\ t \in \mathbb{N}$. The observational functions are $u_i(x_i) = x_i$, the embedding parameters are $d_i = 5$ and $\tau_i = 1$ for $i = 1, \cdots, 5$, and other parameters are $N_\varepsilon = 33$, $e = 0.001$, and $E = 0$. For each network topology, the parameters and the coupling strengths in model (S3.1) are set as follows:

- *Chain topology:* $r_1 = 3.75$, $r_2 = 3.78$, $r_3 = 3.76$, $r_4 = 3.8$, $r_5 = 3.76$, and $\mu_{i+1,i} = 0.07$ for $i = 1, 2, 3, 4$, and all other coupling strengths are 0.
- *Ring topology:* $r_1 = 3.8$, $r_2 = 3.77$, $r_3 = 3.78$, $r_4 = 3.79$, $r_5 = 3.78$, and $\mu_{(i+1) \bmod 5, i} = 0.07$ for $i = 1, 2, 3, 4, 5$ with other coupling parameters being 0.
- *Tree topology:* $r_1 = 3.8$, $r_2 = 3.78$, $r_3 = 3.76$, $r_4 = 3.77$, $r_5 = 3.74$, and $\mu_{i+1,i} = \mu_{i+3,i} = 0.07$ for $i = 1, 2$ with other coupling parameters being 0.

For all the cases, the calculated values of the slope $s_{\boldsymbol{u}_i \hookrightarrow \boldsymbol{u}_j}$ characterizing the causation from x_i to x_j are displayed in Fig. S15(d) and in Figs. 3(c) and 3(d) in the main text. To further characterize the performance of our continuity scaling framework for causal network inferences, we plot the Receiver Operating Characteristics (ROC) curves and calculate the corresponding areas under the ROC curves (AUROCs). As shown in Fig. S15(e), all the AUROC values approach one, demonstrating the superior power of our framework in identifying and quantifying causal interactions in networks.

Continuous time nodal dynamics. We study networks whose nodal dynamics are those of the Lorenz system (S3.3). We also rewrite the system equation here:

$$\begin{aligned} \dot{x}_{i,t} &= \sigma_i(y_{i,t} - x_{i,t}) + \textstyle\sum_{j \neq i} \mu_{ij} x_{j,t}, \\ \dot{y}_{i,t} &= x_{i,t}(\rho_i - z_{i,t}) - y_{i,t}, \\ \dot{z}_{i,t} &= x_{i,t} y_{i,t} - \beta_i z_{i,t}, \end{aligned}$$

where $i = 1, \ldots, N_V,\ t \in \mathbb{R}$. The parameters for the five nodes are taken as: $(\sigma_1, \rho_1, \beta_1) = (10.01, 27.972, 2.668)$, $(\sigma_2, \rho_2, \beta_2) = (9.99, 28.028, 2.6672)$, $(\sigma_3, \rho_3, \beta_3) = (10.012, 27.944, 2.6656)$, $(\sigma_4, \rho_4, \beta_4) = (9.98, 27.9608, 2.66934)$, and $(\sigma_5, \rho_5, \beta_5) = (10.04, 28.056, 2.664)$. The embedding parameters are $d_i = 7$ and $\tau_i = 2\omega$ for $i = 1, \cdots, 5$. The sampling duration is $\omega = 0.016$. Other parameter values are $N_\varepsilon = 33$, $e = 0.001$, and $E = 8$. For each network topology, the coupling parameters and the observational functions are as follows:

- *Chain topology:* $\mu_{i+1,i} = 3$ for $i = 1, \cdots, 4$ with the other coupling strengths being 0, and the observational functions are $u_i(x_i, y_i, z_i) = y_i$ for $i = 1, \cdots, 5$.
- *Ring topology:* $\mu_{(i+1) \bmod 5, i} = 3$ for $i = 1, \cdots, 5$ with all other coupling strengths being 0, and the observational functions are $u_i(x_i, y_i, z_i) = x_i - 0.2\cos(y_i) y_i + 2\sin(z_i)$ for $i = 1, \cdots, 5$.
- *Tree topology:* $\mu_{i+1,i} = \mu_{i+3,i} = 3$ for $i = 1, 2$ with the other coupling strengths being 0, and the observational functions are $u_i(x_i, y_i, z_i) = y_i$ for $i = 1, \cdots, 5$.

In all cases, the values of the slope $s_{\boldsymbol{u}_i \hookrightarrow \boldsymbol{u}_j}$ characterizing the causal interaction from (x_i, y_i, z_i) to (x_j, y_j, z_j) are shown in Figs. S16(a)-16(c). The statistical ROC curves with their AUROCs are displayed in Fig. S16(d), validating the accuracies of the inferred causal interactions in the networks.

E. Test of real-world datasets

1. *Synthetic gene regulatory networks*

This real-world example has been described in the main text. Here we provide necessary additional information on the dataset and important numerical simulation parameters. We consider five different networks consisting of 20 genes, which are randomly selected from five 100-genes-networks for each. One of the network structures is presented in Fig. 5(a) of the main text and the other four structures are shown in Fig. S17. Available are time series of 10 realizations (21 points each) of gene's expressions, which are combined as one of 210 points for each gene in the phase space reconstruction procedure. The

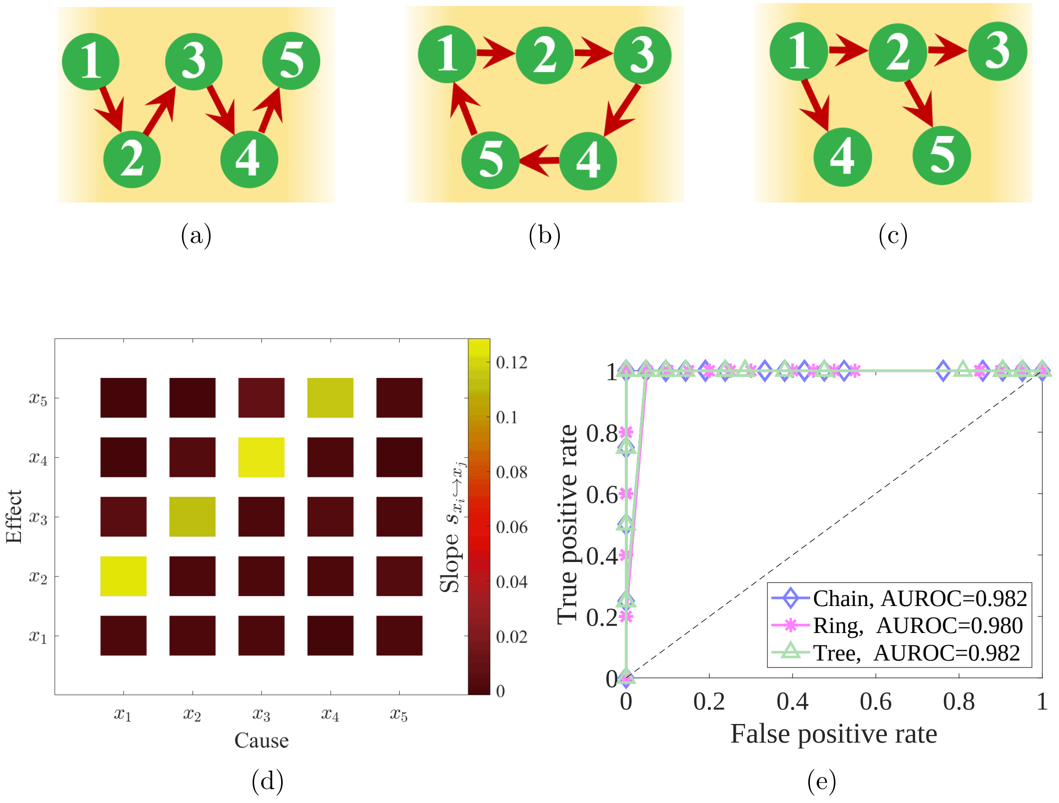

FIG. S15. Inferring causal interactions in networks with the continuity scaling framework. The nodal dynamics are governed by the coupled ecological model of five species. The network structures are (a) chain, (b) ring, and (c) tree. For each case, the results of inferred causation are presented in (d) (chain) as well as in Figs. 3(c) (ring) and 3(d) (tree) of the main text. (e) The corresponding ROC curves and their AUROC values representing high detection accuracies.

embedding dimension and delay time are 2 and 1 respectively. Applying our continuity scaling framework to the time series we obtain the corresponding ROC curves with their AUROC values shown in Fig. 5(b) of the main text.

2. Fishery landings and sea surface temperature data

The California landings data for Pacific sardine and northern anchovy and the sea surface temperature (SST) data are acquired and pre-processed following the supplementary materials of [2].

In each run of continuity scaling, the embedding dimension is 3 and embedding lag is 1 and other parameters are $N_\varepsilon = 33$, $e = 0.001$ and $E = 0$. Each p-value is calculated with 250 random surrogates. Detailed continuity scaling indexes and their p-values are listed in Tab. S12.

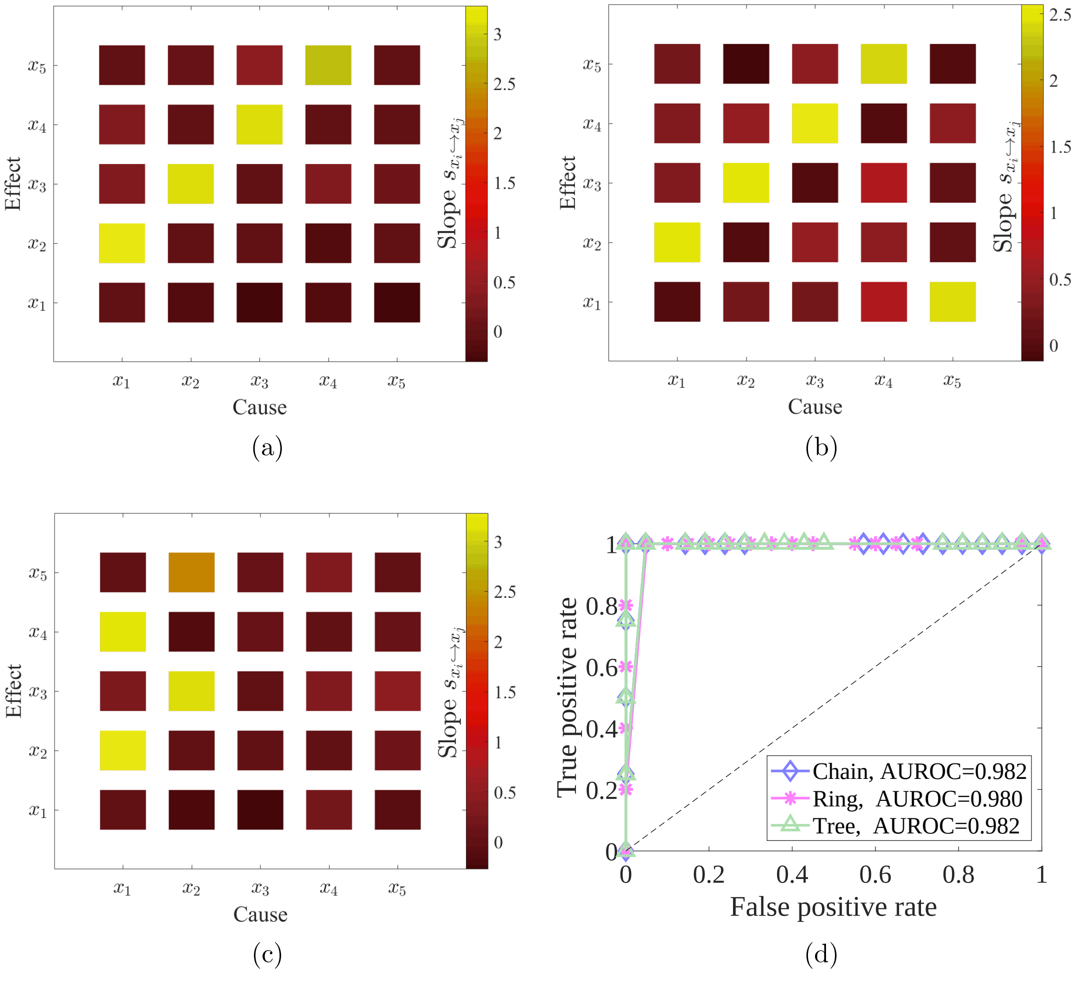

FIG. S16. Inferring causal interactions in networks with continuous time nodal dynamics. The nodal dynamics are described by the Lorenz system (S3.3). The network structures are chain, ring, and tree as in Figs. S15(a)-15(c). The results of causal inference for the three network structures are shown in (a-c), respectively. (d) The corresponding ROC curves and their AUROC values.

3. World COVID-19 pandemic daily cases

We analyze the COVID-19 pandemic data of 19 representitave countries. Time series of daily cumulative confirmed COVID-19 cases from January 22nd 2020 to February 15th 2021 for each country is downloaded from https://datahub.io/core/covid-19#data, and then first-differenced to obtain the time series of daily new confirmed COVID-19 cases. Few negative data points are due to data corruption and are set as zero. Seven-day moving average is applied to the daily new cases time series.

As shown in Fig. S18(a) the pandemic situation in China experiences a remarkable change: severe at first and under control afterwards, and the critical day 100 is suitable to divide these two stages (see below). Thus we split the times series into two segments: day 1 to day 100 (Stage 1) and day 101 to day 391 (Stage 2), and compare the causal effect from China to other countries at these stages. Causation is confirmed pairwisely if the p-value of its continuity scaling index is less than 0.05. The embedding parameters

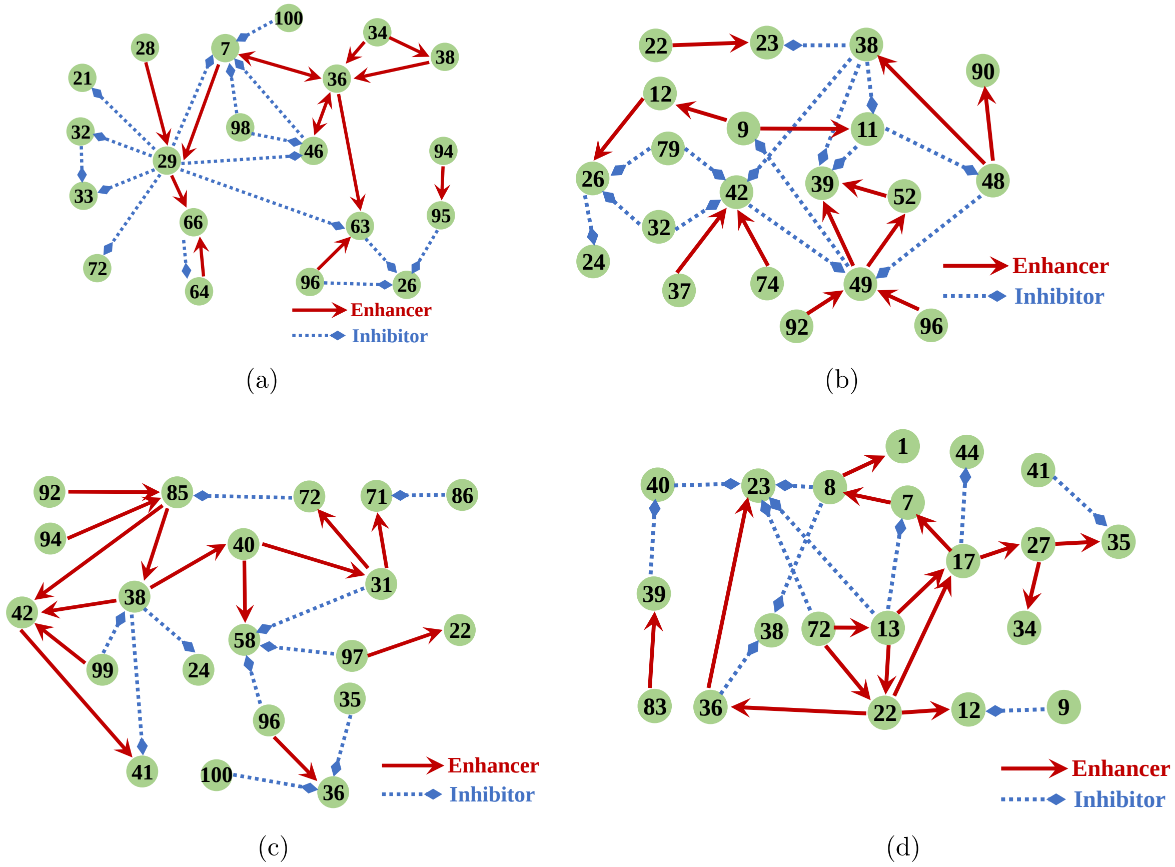

FIG. S17. The four networks together with Fig. 5(a) of the main text, with which our continuity scaling framework is tested.

TABLE S12. Inferring causal interaction among SST and landings data for Pacific sardine and northern anchovy. Slopes of the regression lines of the scaling relations and their p-values are listed.

	slope	p-value ($< \mathbf{0.05}$)
$s_{\text{SST}\hookrightarrow\text{Sardine}}$	**0.0435**	**0.0000**
$s_{\text{Sardine}\hookrightarrow\text{SST}}$	-0.0012	0.6943
$s_{\text{SST}\hookrightarrow\text{Anchovy}}$	**0.0462**	**0.0001**
$s_{\text{Anchovy}\hookrightarrow\text{SST}}$	-0.0239	0.9837
$s_{\text{Sardine}\hookrightarrow\text{Anchovy}}$	0.0025	0.3411
$s_{\text{Anchovy}\hookrightarrow\text{Sardine}}$	**0.0105**	**0.0237**

are $d = 3$ and $\tau = 1$, and other parameters are $N_\varepsilon = 33$, $e = 0.001$ and $E = 0$ for each run. Each p-value is calculated with 200 random surrogates. The detected pairwise causal links are presented in Figs. S18(c)-S18(d) for two stages respectively. Particularly, the detected causal links from China to other countries are depicted in Fig. 7 of the main text. Abbreviation (from ISO 3166 country codes, https://www.iso.org/iso-3166-country-codes.html) or index number denoting each country is listed in Tab. S13.

Additionally, we show that critical day, the last day of Stage 1, can be chosen with moderate freedom and this won't harm the soundness of our result. We split the daily

TABLE S13. Abbreviation and index number used in Fig. 7 of the main text, Fig. S18 and Tab. S14 for each country are listed here.

No.	Abbr.	Country
1	CHN	The People's Republic of China
2	GBR	The United Kingdom of Great Britain and Northern Ireland
3	AUS	The Commonwealth of Australia
4	FRA	The French Republic
5	DEU	The Federal Republic of Germany
6	ITA	The Italian Republic
7	JPN	Japan
8	MYS	Malaysia
9	MEX	The United Mexican States
10	SGP	The Republic of Singapore
11	ZAF	The Republic of South Africa
12	ESP	The Kingdom of Spain
13	SWE	The Kingdom of Sweden
14	CHE	The Swiss Confederation
15	USA	The United States of America
16	KOR	The Republic of Korea
17	BEL	The Kingdom of Belgium
18	NLD	The Kingdom of the Netherlands
19	GRC	The Hellenic Republic

confirmed cases time series of all contries by day D ranging from 94 to 106, and conduct analogous analysis to detect causal links. A directional links labelled by "1" with causation or "0" without causation. We compare the results between critical day D and 100 by counting discrepant results among all links and calculated the proportion. As shown in Fig. S18(b), for all Ds, the proportion of variated results does not exceed 5%, and particularly, the conclusion that no country is under the influence of China at Stage 2 always holds.

For CCM, the embedding dimension is 3 and embedding lag is 1. At the two stages, we use 44, 128 points as library respectively, and calculate CCM index with 30, 90 length time series respectively [2]. Emperical threshold is selected where the largest 1/3 links are identified as positive detections (see [2, 3] for more information on the issues of threshold selection).

The prevalence of COVID-19 pandemic in China exists only at Stage 1, while daily cases at Stage 2 are fewer as shown in Fig. 7 of the main text which are mainly imported cases as reported by the goverment (http://en.nhc.gov.cn/DailyBriefing.html). Therefore minor causal influence from China to other countries can be supposed at Stage 2. However as shown in Tab. S14, at Stage 2 CCM detects a remarkable number of countries still under the influence of China and produces results hard to interpret, lowering its reliablity in widely use.

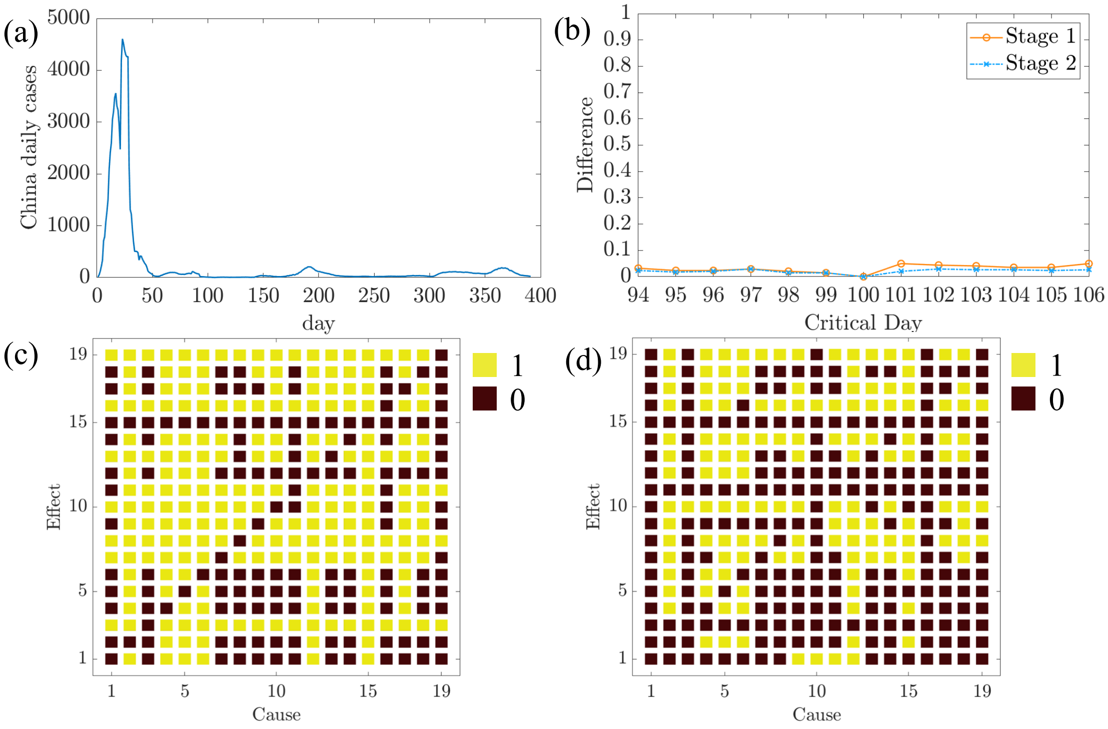

FIG. S18. The 7-day moving averaged daily cases time series of China is plotted in (a). The pairwise causal links detected by continuity scaling from COVID-19 pandemic times series at both stages are illustrated in (c, d). A directional link is colored yellow with causation ("1") or brown without causation ("0"). Each country is represented by its index number listed in Tab. S13. The results of Stage 1 and 2 are presented in (c) and (d) respectively. For critical day, the last day of Stage 1, D ranging from 94 to 106, the proportion of changed causal links compared with critical day 100 is plotted in (b).

TABLE S14. Countries under the influence of China detected by CCM at both stages.

Stage	Countries
1	FRA, DEU, ITA, MYS, ESP, CHE, BEL, NLD
2	GBR, MYS, MEX, ZAF, USA

4. Air pollutants and hospital admission records from Hong Kong

The dataset contains the records of daily air pollutants and of the daily admissions of cardiovascular (Cardio.) disease and respiratory (Resp.) diseases in major hospitals of Hong Kong, China [4, 5] from 1997 to 1999. Every time series contains 1032 points, one for each day. The causal influences of four pollutants, i.e., NO_2, O_3, respirable suspended particulates (Rspar.), and SO_2, on the hospital admissions of Cardio. and Resp. diseases are tested. For all the time series, the embedding dimension and delay time are $d = 4$ and $\tau = 1$, and other parameter values are $N_\varepsilon = 33$, $e = 0.001$, and $E = 0$. The estimated values of the slope $s_{\text{Pollutant}\hookrightarrow\text{Disease}}$ and their p-values are listed in Tab. S15. It can be seen that the instantaneous influences of NO_2 on both Cardio. and Resp. diseases are relatively strong. In addition, the instantaneous impact of O_3 on Cardio. disease and that

TABLE S15. Causal influences, quantified by values of the slope $s_{\text{Pollutant}\hookrightarrow\text{Disease}}$, of four pollutants on the hospital admissions of Cardio. and Resp. diseases, where 300 surrogates are used to test the statistical significance.

	$s_{\text{Pollutant}\hookrightarrow\text{Disease}}$		p-value ($<$ **0.05**)	
Pollutant	Cardio.	Resp.	Cardio.	Resp.
NO_2	**0.5722**	**0.4139**	**0.0000**	**0.0276**
O_3	**0.2427**	0.0856	**0.0000**	0.6529
Rspar.	0.0002	0.0151	0.8404	0.8239
SO_2	-0.0716	**0.1077**	1.0000	**0.0000**

of SO_2 on Resp. disease are also significant, which are highly consistent with previous results [6, 7].

IV. COMPARISONS WITH TYPICAL CROSS-MAP-BASED METHODS

A cross map is defined originally based on the strict correspondence of the time index between the reconstructed manifolds, which is not directly relevant to the existing causal relation, leading to the long-standing question of the use of the information about the effect variable to estimate that of the causal variable. Ref. [8] provides a conceptual explanation from the viewpoint of information flow, which still calls for a rigorous demonstration from the mathematical viewpoint. The definition and the arguments presented in the main text not only provide a resolution to this puzzle in the estimation order but, more significantly, establish a rigorous framework to ascertain reliably and quantify accurately the causal interactions.

We analyze the deficiencies of representative existing cross-map-based methods for causality detection and carry out a comparison study based on benchmark models with known ground truth to demonstrate that our continuity scaling framework is able to overcome the difficulties.

A. Comparison with topological causality

A recently developed technique is based on the concept of topological causality [9]. Let $\boldsymbol{F}$ be a dynamical system on a compact manifold $\mathcal{M}$ with two observational functions: ϕ and ψ, which are smooth functions from $\mathcal{M}$ to $\mathbb{R}$. Suppose further that

$$\boldsymbol{\Phi}(\boldsymbol{x}) = (\phi(\boldsymbol{x}), \phi(\boldsymbol{F}^{\tau}(\boldsymbol{x})), \cdots, \phi(\boldsymbol{F}^{(d-1)\tau}(\boldsymbol{x}))) : \mathcal{M} \to \mathcal{M}_{\phi} = \Phi(\mathcal{M}) \subset \mathbb{R}^d,$$
$$\boldsymbol{\Psi}(\boldsymbol{x}) = (\psi(\boldsymbol{x}), \psi(\boldsymbol{F}^{\tau}(\boldsymbol{x})), \cdots, \psi(\boldsymbol{F}^{(d-1)\tau}(\boldsymbol{x}))) : \mathcal{M} \to \mathcal{M}_{\psi} = \Psi(\mathcal{M}) \subset \mathbb{R}^d$$

are embedding and thus are diffeomorphism to image. Let $\boldsymbol{\phi}_t = (\phi(\boldsymbol{x}_t), \ldots, \phi(\boldsymbol{F}^{(d-1)\tau}(\boldsymbol{x}_t)))$ and $\boldsymbol{\psi}_t = (\psi(\boldsymbol{x}_t), \ldots, \psi(\boldsymbol{F}^{(d-1)\tau}(\boldsymbol{x}_t)))$, where $\boldsymbol{x}_{t+1} = \boldsymbol{F}(\boldsymbol{x}_t)$. Define a cross mapping $\Gamma_{\phi\psi}$: $\mathcal{M}_{\phi} \to \mathcal{M}_{\psi}$ through the correspondence from $\boldsymbol{\phi}_t$ to $\boldsymbol{\psi}_t$ based on the time index t, and further define the extension measure $e^t_{\phi\psi}$ by the product of the singular, larger than one values of the Jacobian matrix of $\Gamma_{\phi\psi}$ evaluated at $\boldsymbol{\phi}_t$. The local topological causality from ψ_t to ϕ_t is defined as $C^t_{\psi\to\phi} = (1 + \ln e^t_{\phi\psi})^{-1}$, and the topological causality from ψ to ϕ

is defined as $C_{\psi\to\phi} = (1 + \langle \ln e^t_{\phi\psi} \rangle_t)^{-1}$, in which $\langle \ln e^t_{\phi\psi} \rangle_t$ represents an average of $\ln e^t_{\phi\psi}$ over all time index t.

We wish to point out that the definition of topological causality is mathematically incomplete and thus cannot resolve the long-standing question of using information about the effect variables to infer that of the causal variables. In particular, eliminating the singular values less than one in its definition is likely to lead to inaccurate evaluation of causality, and the coupling strength may not consistently correspond to the strength of the detected causal interaction. This can be demonstrated through the following continuous time linear dynamical system:

$$\dot{x} = x - \mu y, \quad \dot{y} = y,$$

where μ is the coupling parameter. For simplicity, it is possible to consider topological causality in a local region of the phase space (e.g., a neighborhood of the point $(2, 1)$), since local topological causality index is only evaluated at the investigated point. We consider typical cases here, for example, μ ranging in $[0, 1]$ and (x, y) near $(2, 1)$. Therefore, a one-dimensional system is considered as follows. We localize the phase space by multiplying a real-valued smooth function $\xi : \mathbb{R}^2 \to \mathbb{R}$ ranging in $[0, 1]$ at the right-hand side of the original equations, with ξ supported in an open neighborhood of $(x, y) = (2, 1)$ contained in $\{ (x, y) \,|\, x > y > 0 \}$ and $\xi(2, 1) = 1$. The investigated phase space is given by

$$\begin{aligned} L_\mu = \{ (x, y) = (\alpha(t), \beta(t)) \,|\, &(\alpha(t), \beta(t)) \text{ is a solution of} \\ &\dot{\alpha}(t) = \xi(\alpha, \beta)(\alpha - \mu\beta),\ \dot{\beta}(t) = \xi(\alpha, \beta)\beta,\ \alpha(0) = 2, \beta(0) = 1 \}. \end{aligned}$$

In such a one-dimensional phase space, $\dot{x} = \xi(x, y)(x - \mu y)$, $\dot{y} = \xi(x, y)y$ well defines a dynamical system in it. Because of the localization, coordinate projections of the orbit can indeed reconstruct this system by noting that $\xi(x, y)(x - \mu y) \neq 0$, $\xi(x, y)y \neq 0$ on L_μ, and thus maintain the topological characteristics of the original one-dimensional system: $\dot{x} = x - \mu y$, $\dot{y} = y$.

Considering the new system $\dot{x} = \xi(x - \mu y)$, $\dot{y} = \xi y$ on phase space L_μ. Set the observational functions as x and y, let Γ_{xy} be a cross mapping as the correspondence from $\mathcal{M}_x$ to $\mathcal{M}_y$ based on the time index, and denote as M^t_{xy} the Jacobian matrix of Γ_{xy} at time t. Analytically, we have

$$M^t_{xy} = \frac{\mathrm{d}y}{\mathrm{d}x} = \frac{1}{x/y - \mu}.$$

As defined, e^t_{xy} is the singular value of M^t_{xy} which is larger than one. Typically, for $x,\ y > 0$, if the coupling parameter μ increases from 0 to x/y, M^t_{xy} increases from y/x to $+\infty$, and e^t_{xy} increases from $\max\{1, y/x\}$ to $+\infty$. Consequently, the measure of the topological causality, $C^t_{y\to x} = (1 + \ln e^t_{xy})^{-1}$, decreases monotonously from $(1 + \ln \max\{1, y/x\})^{-1}$ to 0. That is, a larger value of the coupling parameter does not imply a more significant causation detected - a result that is not consistent with the dynamical behavior of the system.

We compare the performance of our continuity scaling framework with topological causality using the algorithms according to the original TC measuring by the index $C_{\psi\to\phi}$. We first consider time series generated by the unidirectionally coupled ecological model (S3.1) with $N_V = 2$, $(r_1, r_2) = (3.8, 3.7)$, $\mu_{12} = 0$, and μ_{21} changing its value

from 0 to 0.05. As shown in Fig. S21, our continuity scaling framework works well in detecting causation for all values of μ_{21}, while the topological causality method fails for small values of μ_{21}.

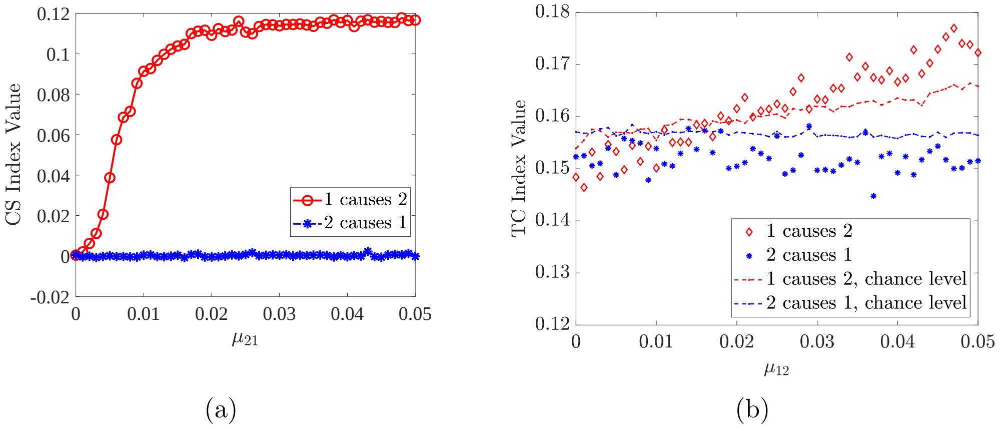

FIG. S19. Results of comparison study using data from the unidirectionally coupled ecological model (S3.1) of two species: $x_1 \hookrightarrow x_2$. (a) Results from our continuity scaling framework with $N_\varepsilon = 33$, $e = 0.001$, and $E = 0$. (b) Results from the topological causality method, where the number of points in the neighborhood is taken to be $k = 15$. For all cases, the observational functions are $u_i(x_i) = x_i$ and the embedding parameters are $d_i = 3$ and $\tau_i = 1$ for $i = 1, 2$.

The second example is the unidirectionally coupled Bernoulli oscillators:

$$\begin{aligned} x_{1,t+1} &= (1.9 \cdot x_{1,t} - \mu_{12} \cdot x_{2,t}) \bmod 1, \\ x_{2,t+1} &= (2.3 \cdot x_{2,t}) \bmod 1, \end{aligned} \tag{S4.1}$$

where the coupling parameter μ_{12} changes its value systematically from 0 to 0.02. Figure S22 presents the results of the comparison study, demonstrating the superior accuracy of our continuity scaling framework for detecting and quantifying causation. The topological causality method reveals completely wrong causation in the weakly coupling regime of small μ_{12} values and exhibits a reversed relation with the increase of μ_{12}.

B. Comparison with convergent cross mapping

We also carry out a comparison study with CCM method based on benchmark models with known ground truth [2]. We calculate the slope values estimated from the linear regression of the convergence curve with increasing length of the library time series in CCM.

We first consider time series generated by the unidirectionally coupled ecological model (S3.1) with $N_V = 2$, $(r_1, r_2) = (3.8, 3.7)$, $\mu_{12} = 0$, and μ_{21} changing its value from 0 to 0.05. As shown in Fig. S21, both our continuity scaling framework and CCM work well in detecting causation for all values of μ_{21}. However, the CCM measure exhibits an increasing level of fluctuations as the coupling parameter is increased.

The second example is the unidirectionally coupled Bernoulli maps:

$$\begin{aligned} x_{1,t+1} &= (1.9 \cdot x_{1,t} - \mu_{12} \cdot x_{2,t}) \bmod 1, \\ x_{2,t+1} &= (2.3 \cdot x_{2,t}) \bmod 1, \end{aligned} \tag{S4.2}$$

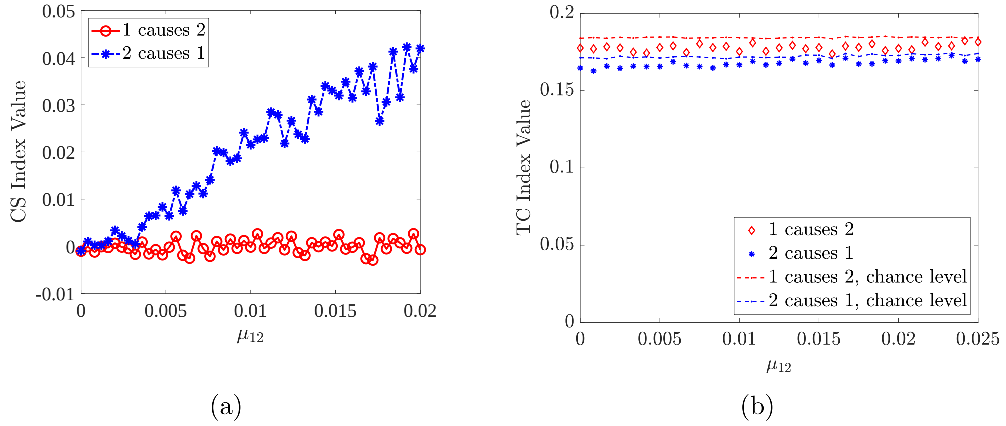

FIG. S20. Results of comparison study using data from the unidirectionally coupled Bernoulli oscillators (S4.2): $x_2 \hookrightarrow x_1$. (a) Results from our continuity scaling framework. (b) Results from the topological causality method. The embedding parameters are $d_i = 4$ and $\tau_i = 1$ for $i = 1, 2$, and the observational functions and other parameter values are the same as those in Fig. S21.

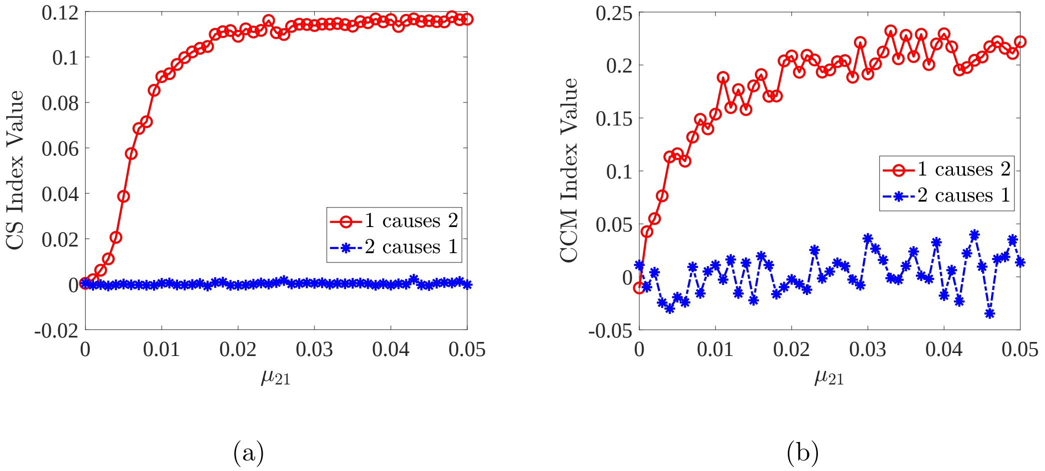

FIG. S21. Results of comparison study using data from the unidirectionally coupled ecological model (S3.1) of two species: $x_1 \hookrightarrow x_2$. (a) Results from our continuity scaling framework with $N_\varepsilon = 33$, $e = 0.001$, and $E = 0$. (b) Results from the CCM method. For all cases, the observational functions are $u_i(x_i) = x_i$ and the embedding parameters are $d_i = 3$ and $\tau_i = 1$ for $i = 1, 2$.

where the coupling parameter μ_{12} changes its value systematically from 0 to 0.02. Figure S22 presents the results of the comparison study, demonstrating the superior accuracy of our continuity scaling framework for detecting and quantifying causation. The CCM method fails for most values of the coupling parameter. The reason for the failure lies in that the Bernoulli maps have uniformly expanding properties with all positive Lyapunov exponents, leading to the sensitivity of detection on the pre-selected radius of the neighborhoods. Increasing the time series length in the CCM method does not provide

a resolution. In contrast, the continuity scaling between the dynamically contracting neighborhoods is key to determining the accuracy of causation detection.

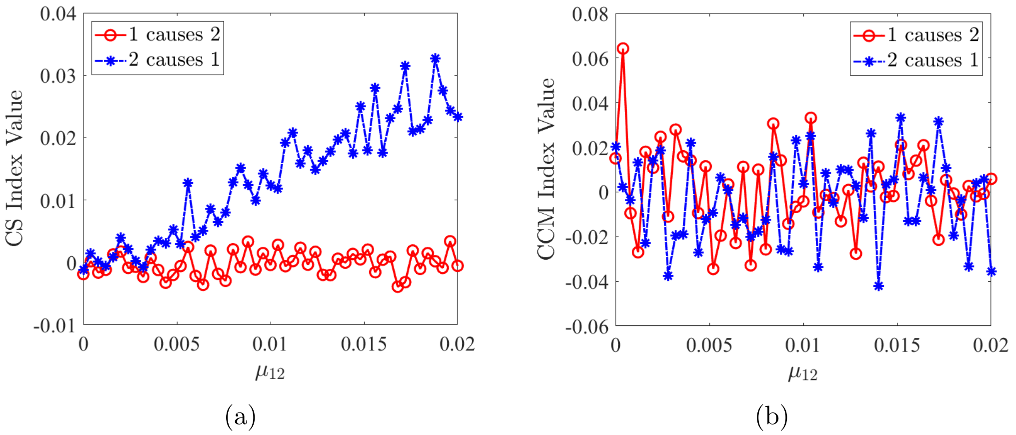

FIG. S22. Results of comparison study using data from the unidirectionally coupled Bernoulli maps (S4.2): $x_2 \hookrightarrow x_1$. (a) Results from our continuity scaling framework. (b) Results from the CCM method. The embedding parameters are $d_i = 5$ and $\tau_i = 1$ for $i = 1, 2$, and the observational functions and other parameter values are the same as those in Fig. S21.

Though greatly inspired by the cross-map-based methods, we still summarize the essential differences between our CS framework and the cross-map-based methods here. While the correspondence techniques using the time indexes in the traditional cross-map-based methods and our CS framework are almost the same, they differ in one-step time index and also in using a [DD] condition. These differences follow from the consideration and quantification of continuity for different maps (Φ or f). These differences also yield the above example where our framework outperforms the CCM method even using sufficiently large embedding dimensions. Precisely, in the traditional cross-map-based techniques, function properties including continuity regarding the cross-map $Y_t = \Phi(X_t)$ (when Y drives X) are fully and systematically investigated. Thus, correspondence technique using the simultaneous points is taken into account. But we consider the causality using the original dynamical systems, not the cross-map Φ. Particularly, when turning eyes from the cross-map to the original iteration/flow, i.e., $x_{t+1} = f(x_t, y_t)$, correspondence between y_t and x_{t+1} is considered, with one-step time difference naturally arising. This minor difference in implementation in fact is induced by essential difference in changing object of study. Secondly, the above consideration requires the key Condition [DD], which is only needed when considering the original iteration/flow and whose universality is demonstrated in section II. Thirdly, the idea of the CS framework is that causality is reflected in the continuity scaling relationship of the original iteration/flow, i.e., in the scaling relation between changing sizes of the $\varepsilon_{x_{t+1}}$ and δ_{y_t} neighborhoods, which leads to the failure of the CCM method using pre-selected/fixed radius of the neighborhoods in the example of coupled Bernoulli maps, with uniformly expanding properties. These points illustrate the importance of directly investigating the continuity of the original iteration/flow f rather than the cross-map Φ.

In addition, our proposed CS framework has different object of study with all the existing methods and holds essentially different theoretical basis. In fact, the existing causation detection methods, referred to in the main text, can be roughly classified into the

following categories: Granger causality based methods, entropy based methods, and cross-map-based methods. While a complete summarization study of the Granger causality based methods and entropy based methods can be referred to many literatures [3, 10], the above comparisons with typical cross-map-based method demonstrate their differences with our CS framework.

---

[1] Eckmann, J.-P. & Ruelle, D. Ergodic theory of chaos and strange attractors. *Reviews of Modern Physics* **57**, 617–656 (1985). doi: 10.1103/RevModPhys.57.617.

[2] Sugihara, G. *et al.* Detecting causality in complex ecosystems. *Science* **338**, 496–500 (2012). doi: 10.1126/science.1227079.

[3] Leng, S. *et al.* Partial cross mapping eliminates indirect causal influences. *Nature Communications* **11**, 1–9 (2020). doi: 10.1038/s41467-020-16238-0.

[4] Fan, J., Zhang, W. *et al.* Statistical estimation in varying coefficient models. *Annals of Statistics* **27**, 1491–1518 (1999). doi: 10.1214/aos/1017939139.

[5] Wong, T. W. *et al.* Air pollution and hospital admissions for respiratory and cardiovascular diseases in hong kong. *Occupational and Environmental Medicine* **56**, 679–683 (1999). doi: 10.1103/PhysRevE.96.012221.

[6] Ma, H. *et al.* Detection of time delays and directional interactions based on time series from complex dynamical systems. *Physical Review E* **96**, 012221 (2017).

[7] Milojevic, A. *et al.* Short-term effects of air pollution on a range of cardiovascular events in england and wales: case-crossover analysis of the minap database, hospital admissions and mortality. *Heart* **100**, 1093–1098 (2014). doi: 10.1136/heartjnl-2013-304963.

[8] Paluš, M., Krakovská, A., Jakubík, J. & Chvosteková, M. Causality, dynamical systems and the arrow of time. *Chaos: An Interdisciplinary Journal of Nonlinear Science* **28**, 075307 (2018). doi: 10.1063/1.5019944.

[9] Harnack, D., Laminski, E., Schünemann, M. & Pawelzik, K. R. Topological causality in dynamical systems. *Physical Review Letters* **119**, 098301 (2017). doi: 10.1103/PhysRevLett.119.098301.

[10] Edinburgh, T *et al.* Causality indices for bivariate time series data: A comparative review of performance. *Chaos: An Interdisciplinary Journal of Nonlinear Science* **31**, 083111 (2021). doi: 10.1063/5.0053519.
